# Quantifying Urban Morphology-Induced Uncertainty in Urban Meteorology and Heat Stress Simulations in Southern California

**DOI:** 10.1029/2025jd045318

**Published:** 2026-02-21

**Authors:** Hao Hu, Xinyi Zhang, Cenlin He, Ellis Fertig, Reza Zarrin, Soroush E. Neyestani, Jiachen Zhang

**Affiliations:** 1 Department of Civil and Environmental Engineering, University of Southern California, Los Angeles, CA, USA; 2 NSF National Center for Atmospheric Research (NCAR), Boulder, CO, USA

## Abstract

Accurate representation of land use and urban morphological parameters (UMPs), particularly building height, road width, and roof width, is critical for urban climate/weather modeling. The Weather Research and Forecasting (WRF) coupled with the Single-Layer Urban Canopy Model (SLUCM) has been widely used; however, few studies have quantified urban modeling uncertainties associated with UMPs in Los Angeles, a key metropolitan area. This study assesses the impact of UMPs on WRF–SLUCM simulations in Los Angeles and quantifies UMP-induced uncertainties in 2-m air temperature (*T*_2_), relative humidity (RH), wind speed and other outputs by integrating Polynomial Chaos Expansion, Sobol sensitivity analysis, and Monte Carlo methods. We apply urban Local Climate Zone (LCZ) land use data and find that incorporating accurate UMPs based on Los Angeles County improves wind speed simulations compared with default LCZ UMPs. Uncertainty analyses reveal strong sensitivities of urban meteorology to 50% UMP perturbation. The resulting average standard deviations of 2-m air temperature are 0.02 K (day) and 0.23 K (night), while those of urban canyon temperature are 0.51 K (day) and 1.03 K (night). Wind speed uncertainties are also notable, reaching 0.42 m/s (day) and 0.27 m/s (night). Among UMPs, building height has the strongest influence on urban T2, RH, and wind speed. Furthermore, uncertainties in urban meteorology propagate into heat stress estimates, where different indices show different spatial patterns of uncertainty. These findings underscore the importance of accurately representing UMPs in urban climate/weather simulations and their implications for assessing urban heat stress.

## Plain Language Summary

Computational models are widely used to study how city features, such as tall buildings or wide streets, affect local weather and heat stress. To keep these models practical, researchers often make simplifications about building and road geometry. However, these simplifications can introduce uncertainty into results, and past studies have not systematically evaluated the magnitude of those uncertainties in Southern California. In this study, we use a coupled weather-urban model to quantify how changes in urban geometry such as building height, roof width, and road width affect local wind, temperature, humidity, and heat stress. We find that these features strongly shape air movement, temperature, and humidity in street canyons, and the uncertainties from building geometry also carry over into different urban heat stress measures. Importantly, the amount and location of uncertainty can vary depending on the heat stress index is used. Our results show that including more accurate details about urban geometry can make model predictions more reliable. This is especially important for Southern California, where extreme heat is already a serious concern for public health.

## 1. Introduction

Over half of the world’s population now resides in cities, and this proportion is projected to reach approximately 68% by 2050 (Affairs, 2019). Rapid urbanization is frequently associated with various environmental and climate issues, including the urban heat island effect (Tam et al., 2015) and risks to public health (Heaviside et al., 2017). To assess the climate impacts of urbanization and potential mitigation strategies, it is important to have a more comprehensive understanding of the interactions between urban surfaces and the atmosphere, and to enhance their representation in atmospheric models.

The Weather Research and Forecasting (WRF) model coupled with the Single-Layer Urban Canopy Model (SLUCM) has been extensively employed to investigate urban climate issues (Zhu & Ooka, 2023), including land-use change (Yao & Huang, 2023) and anthropogenic heat release (Xu et al., 2024; Y. Zhao et al., 2021), as well as strategies for heat mitigation (Schlaerth et al., 2023; Zhang et al., 2018). The SLUCM model represents urban geometry as infinitely long street canyons to simulate the physical interactions between the atmosphere and urban surfaces (Kusaka et al., 2001; Masson, 2000). Previous studies show that the accuracy of land-use data influences the performance of WRF-SLUCM simulations in cities (F. Chen et al., 2014; G. Chen et al., 2016; S. Zhao et al., 2024). In Southern California, the National Land Cover Database is typically used for land use input (Y. Li et al., 2019; Vahmani & Ban-Weiss, 2016; Xu et al., 2024; Zhang et al., 2018), categorizing urban areas into three classifications (Homer et al., 2015). In contrast, the Local Climate Zone (LCZ) land use classification data set provides a more detailed description of urban areas by dividing them into 10 types based on their form, function, and location (Demuzere et al., 2023; Stewart & Oke, 2012). Recent studies have increasingly incorporated LCZ data into WRF-SLUCM for urban climate simulations (Ding & Chen, 2024; Smithson & Adams, 2024), including the application in the Southern California region (Herrmann & Gutheil, 2022).

In addition to land use classification, the performance of WRF-SLUCM simulations is also influenced by urban morphological parameters (UMPs), such as building height, road width, and roof width (B. Chen et al., 2021). UMPs affect urban simulations by altering surface radiative energy balance and aerodynamic roughness. For example, the ratio of building height to road width (i.e., street canyon aspect ratio) modulates longwave-radiation trapping and shortwave-radiation shading (Hang & Chen, 2022; S.-H. Lee et al., 2016; Theeuwes et al., 2014). Additionally, a greater building height increases aerodynamic roughness length and drag, thereby reducing near-surface wind speed (Hang & Chen, 2022; Liu et al., 2020). Southern California experiences persistent non-attainment of air-quality standards (South Coast AQMD, 2022) and severe urban heat stress (Hulley et al., 2020), which requires evaluation of solutions to these challenges (Epstein et al., 2017). Many studies have employed the WRF-SLUCM model to investigate the impacts of urbanization and the effects of mitigation strategies (Table S1 in Supporting Information S1). However, most of these simulations rely on default non-region-specific UMPs and the National Urban Database and Access Portal Tool (NUDAPT) data (Ching et al., 2009) developed prior to 2010, which only cover a portion of the region’s urban areas. Additionally, the extent to which UMPs influence WRF-SLUCM simulations over Southern California remains insufficiently understood.

Sensitivity analyses have been conducted in previous studies to examine the influence of urban morphology on simulated surface temperature, wind speed, and radiative fluxes (Theeuwes et al., 2014; Z. Wang et al., 2011; M. Yu et al., 2021). Furthermore, recent studies have investigated the sensitivity of the universal thermal climate index (Yang et al., 2024) and heat index (HI) to UMPs (Kotharkar et al., 2023). However, systematic quantification of uncertainties resulting from UMPs is still limited, particularly in online coupled WRF-SLUCM simulations due to large computational costs. Although recent data sets have developed enhanced urban morphology data sets (Liao et al., 2024), WRF-SLUCM simulations by default still employ predefined UMPs with nontrivial uncertainty. Thus, rigorous uncertainty analysis is critical for identifying the most influential UMPs, constraining model errors, and ultimately enhancing the robustness and applicability of WRF-SLUCM simulations for urban planning and climate adaptation.

Uncertainty quantification of models is usually conducted by applying probabilistic approaches, such as the Monte Carlo method and its variants (Zhang, 2021), due to their simplicity and generality. However, the Monte Carlo method and its variants require numerous model trials, making it challenging to quantify the uncertainty of WRF simulations that are very computationally expensive. Previous studies reduced the computational costs by shortening the duration of simulations, lowering the spatial resolution of the model (Moosavi et al., 2021), or using offline SLUCM experiments (Z. Wang et al., 2011). Alternatively, the Polynomial Chaos Expansion (PCE) method (Palar et al., 2018), which requires drastically fewer simulations, has been employed to quantify the uncertainty of WRF simulations, such as qualifying uncertainty in precipitation (Du et al., 2022) and wind speed (Jin et al., 2019) arising from the parametric uncertainty in the physics schemes. However, to our knowledge, no study has applied PCE to quantify the uncertainty of online coupled WRF-SLUCM simulations associated with UMPs.

Overall, quantifying uncertainties in WRF-SLUCM simulations involves substantial computational resources, and systematic assessments of the uncertainties induced by UMPs remain lacking. To address these research gaps, we derive LCZ-based UMPs tailored explicitly for the LA region to enhance urban climate modeling accuracy and evaluate how customized UMPs affect the performance of WRF-SLUCM simulations. To quantify the uncertainties associated with UMPs, we established a comprehensive uncertainty quantification framework integrating PCE and the Monte Carlo methods. Additionally, we performed a global sensitivity analysis utilizing Sobol indices derived from the PCE results to pinpoint the most influential UMP parameters. We then apply this integrated framework to assess uncertainties in both meteorological outputs and heat stress indicators resulting from variations in UMPs. To our knowledge, this is the first study to examine how land-use classification and UMPs affect WRF-SLUCM simulations over Southern California and the first to systematically quantify UMP-induced uncertainties in meteorological variables and heat-stress indicators in the region. Our findings offer crucial insights into model uncertainties, highlighting the need to accurately characterize UMPs for reliable urban climate modeling and effective heat stress assessments.

## 2. Methods

### 2.1. Study Area

**Figure 1.**
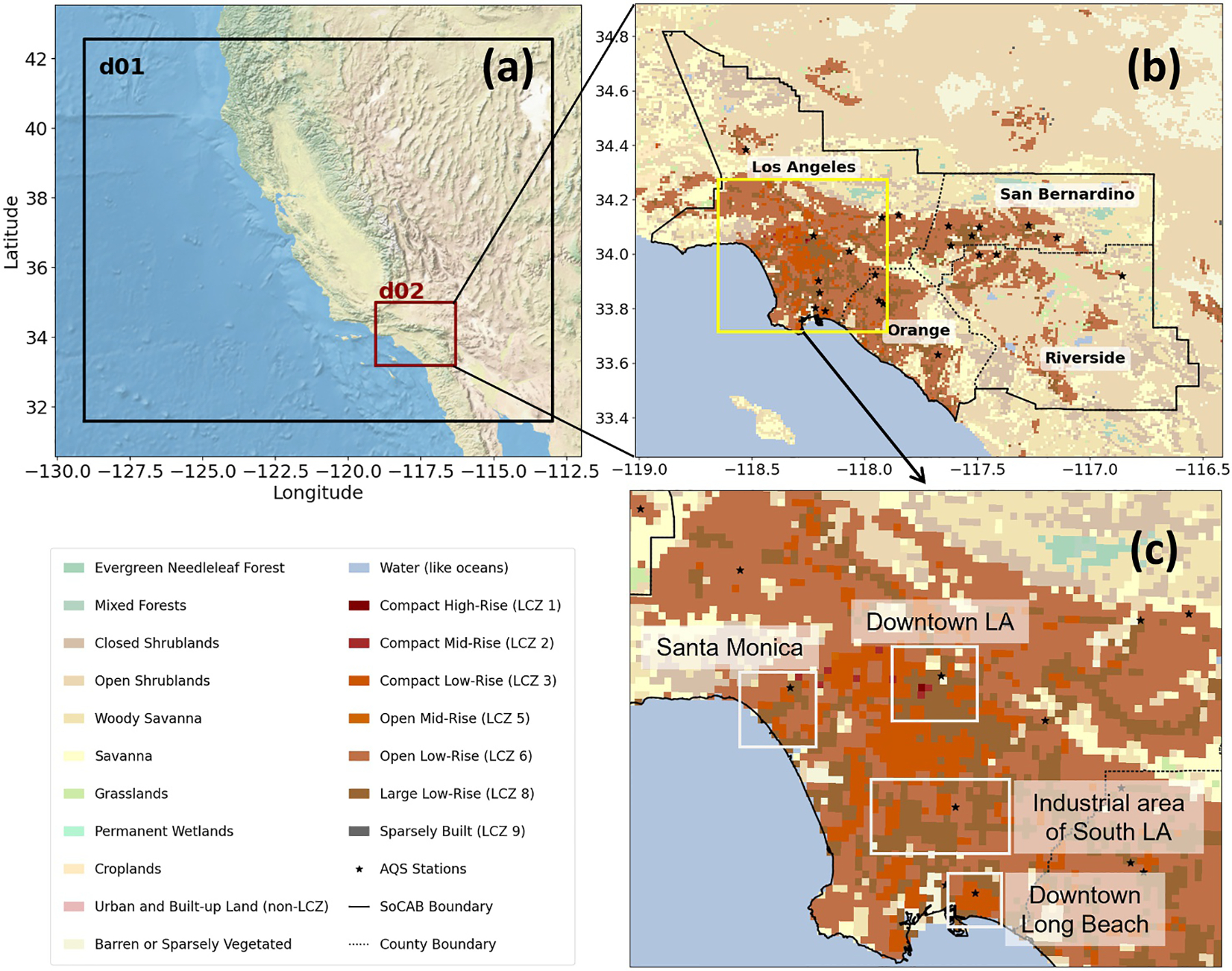
(a) The two nested model domains used in the WRF-SLUCM simulations (base map: World Street Map from ArcMap). (b) Map of land use types from MODIS, urban Local Climate Zone classifications, and locations of Air Quality System observation stations (black star symbols) within the South Coast Air Basin. (c) Zoomed-in view of panel (b) over Los Angeles County.

Figure 1b shows the study region, the South Coast Air Basin (SoCAB), including Los Angeles, Orange, San Bernardino, and Riverside Counties, which is one of the largest metropolitan areas in the world. Los Angeles County alone spans more than 4,000 square miles and is among the most populous regions in the United States, with roughly 10 million residents across 88 cities, including the City of Los Angeles (U.S. Census Bureau, 2022). A zoomed-in view of Los Angeles County is shown in Figure 1c. SoCAB is a geographically diverse region that includes coastal plains, mountain ranges, and densely urbanized land. Southern California also experiences substantial environmental challenges and has served as a testbed for numerous climate and air-quality modeling studies, making this region a particularly valuable case for investigating urban climate processes. Figure 1b highlights the detailed land use and land cover classifications within the study area, where the LCZ categories represent urban areas.

### 2.2. Model Configuration

This study uses the WRF model with the Advanced Research WRF core, version 4.6.1. The simulation is configured with two nested domains in a Lambert conformal projection (Figure 1a). The outer domain (d01) has a horizontal resolution of 4 km and encompasses the entire state of California, providing boundary conditions for the inner domain. The inner domain (d02) covers the SoCAB region at a 1 km resolution, which is the focus of this study (Figure 1b). The model integration time step is 24 s for the parent domain and a parent-to-child time step ratio of 3:1 to reduce simulation cost. This configuration satisfies the Courant–Friedrichs–Lewy (CFL) criterion and produces stable simulations (Courant et al., 1928; Skamarock et al., 2019). A total of 45 terrain-following vertical eta levels is employed, extending from the surface up to 50 hPa. The initial conditions of d01 and d02, as well as the boundary condition of d01, are based on the National Centers for Environmental Prediction (NCEP) Global Data Assimilation System final analysis data at a 0.25° resolution and a temporal resolution of 6 hr (Commerce, 2015). No nudging techniques are applied in our simulations, as our objective is to test the sensitivity of meteorological variables to UMPs without interference from nudged boundary conditions that would confound the sensitivity.

Our baseline model setup uses the following physics schemes: the Morrison 2-moment for microphysics scheme (Morrison et al., 2009), the Kain–Fritsch Cumulus Potential for cumulus parameterization scheme (Berg et al., 2013), the RRTMG Shortwave and Longwave for radiation scheme (Iacono et al., 2008), the Quasi–normal Scale Elimination for planetary boundary layer and surface layer physics scheme (Sukoriansky et al., 2005), and the Unified Noah land surface model for land surface model scheme (Tewari, 2004). The Kain–Fritsch cumulus potential scheme is activated for the d01 and disabled for the d02 to allow for explicit resolving of convection.

Model output variables are saved at an hourly interval for analysis. In addition to the above baseline configuration, we further test 24 different combinations of WRF physics parameterization schemes to evaluate their impact on model performance. The full list of all physics schemes is provided in Table S2 in Supporting Information S1, and the combinations of the 24 physics scheme tests are detailed in Table S3 in Supporting Information S1. Detailed evaluation results for each physics configuration are provided in Figure S1, which informs the selection of our baseline physics schemes. Detailed evaluation methods and metrics are provided in Text S2 in Supporting Information S1.

For simulations assessing the influence of UMPs on model performance in SoCAB, we run the model for 16 days, from 00:00 UTC on 1 July to 00:00 UTC on 17 July 2019, to represent summertime climate. For quantifying the uncertainties associated with UMPs and physics schemes, we run more than 100 simulations, each of 8 days (00:00 UTC on 1 July to 00:00 UTC on 8 July 2019). In all simulations, the first 48 hr are discarded as spin-up.

### 2.3. Single Layer Urban Canopy Model (SLUCM)

We use the LCZ default data set to specify the urban fraction across the d02 for different land use types; urban fraction refers to the proportion of each grid cell covered by impervious urban surfaces (i.e., roofs, walls, paved roads). For the urban portion of grid cells in the inner domain d02, SLUCM (Kusaka et al., 2001) is employed to represent the urban canopy as a two-dimensional street canyon, where UMPs (i.e., building height, road width, and roof width) are explicitly defined, and separate energy balance equations are solved for roofs, walls, and roads. Building height and road width influence the amount of shortwave radiation reaching paved roads and the emissions of longwave radiation within the canyon.

SLUCM calculates urban canopy temperature (*T*_C_) as an aggregated temperature, which is derived from the balance of sensible heat flux from the canyon to the lowest atmosphere. Previous studies have indicated that, for urban areas, *T*_C_ represents temperature at the thermal roughness length above the displacement height (Lemonsu et al., 2004; Qin et al., 2023). In contrast, grid-cell average 2-m air temperature (*T*_2_) is diagnosed by the surface-layer scheme using the Monin-Obukhov similarity theory and temperature from the lowest atmospheric model level, representing an area-weighted average of urban and non-urban contributions (F. Chen et al., 2011).

The distinction between *T*_2_ and *T*_C_ has been discussed in the study by Qin et al. (2023), and both variables can be relevant for urban climate. *T*_2_ is often used for model evaluation and heat impact assessment, as it is a standard output of the WRF model. *T*_C_ has also been applied in exploring the uncertainty associated with its calculation methods (D. Li et al., 2024). In this study, we modify the SLUCM code to output its diagnostic variables, including *T*_C_, urban net shortwave radiation flux (*SW*_NET_), urban net longwave radiation flux (*LW*_NET_), and urban net radiation flux (*R*_NET_), to investigate the processes within SLUCM.

### 2.4. Assessment of the Impact of UMPs

To enhance the accuracy of the urban morphology representation of Southern California, we derive UMPs using high-resolution data from the Los Angeles Region Imagery Acquisition Consortium data set (LARIAC, County of Los Angeles, 2014). The methodology for calculating these parameters is described in Text S1 in Supporting Information S1. These improved parameter values are incorporated into the input tables of the SLUCM model, while default values are used for the other SLUCM parameters. We conduct two WRF-SLUCM simulations to assess the effects of UMPs in Southern California, LCZ_LA and LCZ_Default configurations. For the LCZ_LA configuration, we use customized UMPs based on the LA County. For the LCZ_Default configuration, we use the default UMPs of WRF-UCM, which represent general urban conditions (Stewart & Oke, 2012). These simulations allow a direct comparison between default versus customized UMP settings with LCZ land use data as input. The values of UMPs used in the two simulations are listed in Tables S4 and S5 in Supporting Information S1. All other SLUCM parameters are set to their default values.

To evaluate the capability of WRF-SLUCM in simulating local near-surface meteorological variables and to compare their performance under different configurations, we utilize 2-m air temperature (*T*_2_), relative humidity (RH), and wind speed data from the US EPA’s Air Quality System (AQS) database (US EPA, 2012). As we focus on urban climate, we only evaluate our model performance against AQS observation sites in urban areas (Figure 1b) using metrics including mean bias (MB), mean absolute error (MAE), root mean square error (RMSE), and the coefficient of determination (*R*^2^). Detailed calculation methods are provided in Text S2 in Supporting Information S1.

### 2.5. Uncertainty Quantification Method

We systematically assess the uncertainties of WRF-SLUCM simulated meteorological variables and energy fluxes induced by variations in UMPs, including building height, road width, and roof width. Each UMP parameter is multiplied by a scaling factor ranging from 0.5 to 1.5, reflecting a ±50% perturbation around our customized UMPs for the LCZ_LA configuration. Note that we also apply the scaling factor of building height to the building height standard deviation (SD), ensuring consistent scaling of building geometry in our analysis. Text S3 in Supporting Information S1 discusses the rationale behind selecting the 50% perturbation, which is constrained by observations. The uncertainty of the following WRF-SLUCM output variables is assessed: *T*_2_, *T*_C_, RH, wind speed, planetary boundary layer height (PBLH), *SW*_NET_, *LW*_NET_, and *R*_NET_.

To reduce the computational cost required by uncertainty quantification, we employ the PCE method. PCE approximates the model response to input variables using a spectral expansion with orthogonal polynomials, which drastically reduces the number of required samples compared to brute-force Monte Carlo approaches (Palar et al., 2018). As a result, PCE significantly lowers computational cost while effectively capturing both linear and nonlinear parameter–output relationships.

In our study, we first use the Latin Hypercube Sampling method, which ensures a stratified and efficient exploration of the entire parameter space, to create 100 UMP sets and run 100 WRF-SLUCM simulations with the parameter sets. We then train the surrogate model with 100 simulation outputs to represent the model behavior. Considering the sample size, we select the best-fitting model among the PCE methods with polynomial degrees 1 to 3 only. When training on each grid cell, the computational cost is substantial; therefore, the polynomial degree is fixed at 3 for every grid cell. After obtaining the PCE coefficients, statistical metrics and global sensitivity indices (Sobol indices) are analytically derived, providing clear insights into uncertainty sources and propagation. Furthermore, we perform Monte Carlo simulations based on the PCE surrogate model to generate the output distribution. Additionally, the interquartile range (IQR) and median absolute deviation are calculated from the Monte Carlo simulations as well to reduce biases caused by extreme values or skewed distributions. Detailed methodology can be found in Text S4 and S5 in Supporting Information S1.

While the primary purpose of this study is to quantify the uncertainty induced by UMPs, we also quantify the uncertainty due to the choice of urban physics schemes by computing the SD of WRF outputs from 24 physics scheme combinations (Table S2 in Supporting Information S1). Note that, except for the physics schemes being tested, other model options and parameters are fixed to the LCZ_LA configuration. The primary goal of these simulations is to compare uncertainties arising from physics scheme selections with those induced by UMPs.

### 2.6. Heat Stress Indicators

This study further investigates the impact of UMP uncertainties on four widely used representative heat stress indicators. These indicators include the Environmental Stress Index (ESI) (Moran & Epstein, 2006), HI (Buzan et al., 2015), Net Effective Temperature (NET) (P. W. Li & Chan, 2000), and Wet Bulb Globe Temperature (WBGT) (Budd, 2008). The calculation methods for these four heat stress indicators are summarized as Equations 1–4. Note that WBGT is calculated using a simplified algorithm from WRF (Yamamoto et al., 2018).

(1) $$\text{ESI}=0.63\;T_{\text{d}}-0.03\;\text{RH}+0.002\;\text{S}+0.0054\;T_{\text{d}}\;\text{RH}-\frac{0.073}{0.1+\text{S}}$$

(2) $$\text{HI}=-42.379+2.04901523\;T_{\text{d}}+10.14333127\;\text{RH}-0.22475541\;T_{\text{d}}\;\text{RH}-0.00683783\;T_{\text{d}}^{2}-0.05481717\;{\text{RH}}^{2}+0.00122874\;T_{\text{d}}^{2}\;\text{RH}+0.00085282\;T_{\text{d}}\;{\text{RH}}^{2}-0.00000199\;T_{\text{d}}^{2}\;{\text{RH}}^{2}$$

(3) $$\text{NET}=37-\frac{37-T_{\text{d}}}{\left[0.68-0.0014\;\text{RH}+\frac{1}{1.76+1.4\;{\text{U}}^{0.75}}\right]}-0.29\;T_{\text{d}}(1-0.01\;\text{RH})$$

(4) $$\text{WBGT}=0.7\;T_{\text{w}}+0.2\;T_{\text{g}}+0.1\;T_{\text{d}}\;T_{\text{g}}=\left\{\begin{array}{l} T_{\text{d}}+12.1+0.0067\;\text{S}-2.40\;{\text{U}}^{0.5}(\text{S}>400)\\ T_{\text{d}}-0.3+0.0256\;\text{S}-0.18\;{\text{U}}^{0.5}\left(\text{S}\leq 400\right) \end{array}\right.$$

Where *T*_d_ is dry-bulb temperature (°C), corresponding to the WRF output variable *T*_2_. RH is relative humidity (%), which is calculated from 2-m specific humidity, *T*_2_, and surface pressure. S is surface downward solar radiation (W/m^2^). U is total wind speed (m/s), which can be calculated using the WRF output variables U10 and V10 as $\sqrt{\text{U}10^{2}+\text{V}10^{2}}$ is the wet-bulb temperature (°C), calculated using the approximate formula proposed by Stull (2011), and *T*_g_ is the globe temperature (°C).

## 3. Results

### 3.1. Evaluation of Model Performance

**Figure 2.**
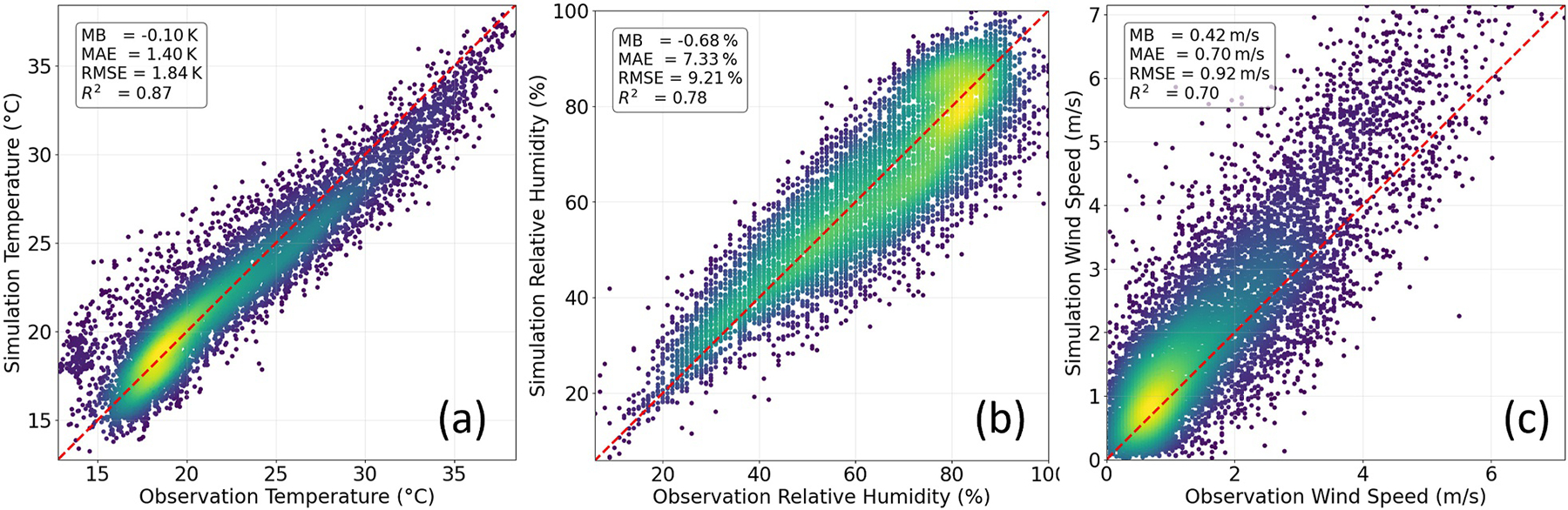
Simulated versus observed hourly (a) 2-m air temperature, (b) relative humidity, and (c) 10-m wind speed in summer. Each dot represents a paired hourly observation and simulation value, with brighter colors indicating higher data density.

Figure 2 shows the evaluation of the baseline WRF-SLUCM simulation (LCZ land use data with customized UMPs) against 24 AQS stations located within SoCAB. Model simulations of *T*_2_, RH, and wind speed perform well in summer, as evidenced by evaluation metrics and scatter plots in Figure 2. For *T*_2_ simulations, MB remains below 0.5 K, and the MAE is less than 2.0 K, aligning with recommended thresholds for WRF model performance reported in the literature (Emery et al., 2017). Similarly, RH and wind speed simulations show strong performance, with low MAE and RMSE and high correlation with observations. Overall, our model configuration achieves performance comparable to or better than previous WRF urban climate studies (Gutiérrez et al., 2015; Kumar et al., 2024; Qian et al., 2025; Vahmani et al., 2022).

### 3.2. Impact of Urban Morphological Parameters on Model Simulations

**Figure 3.**
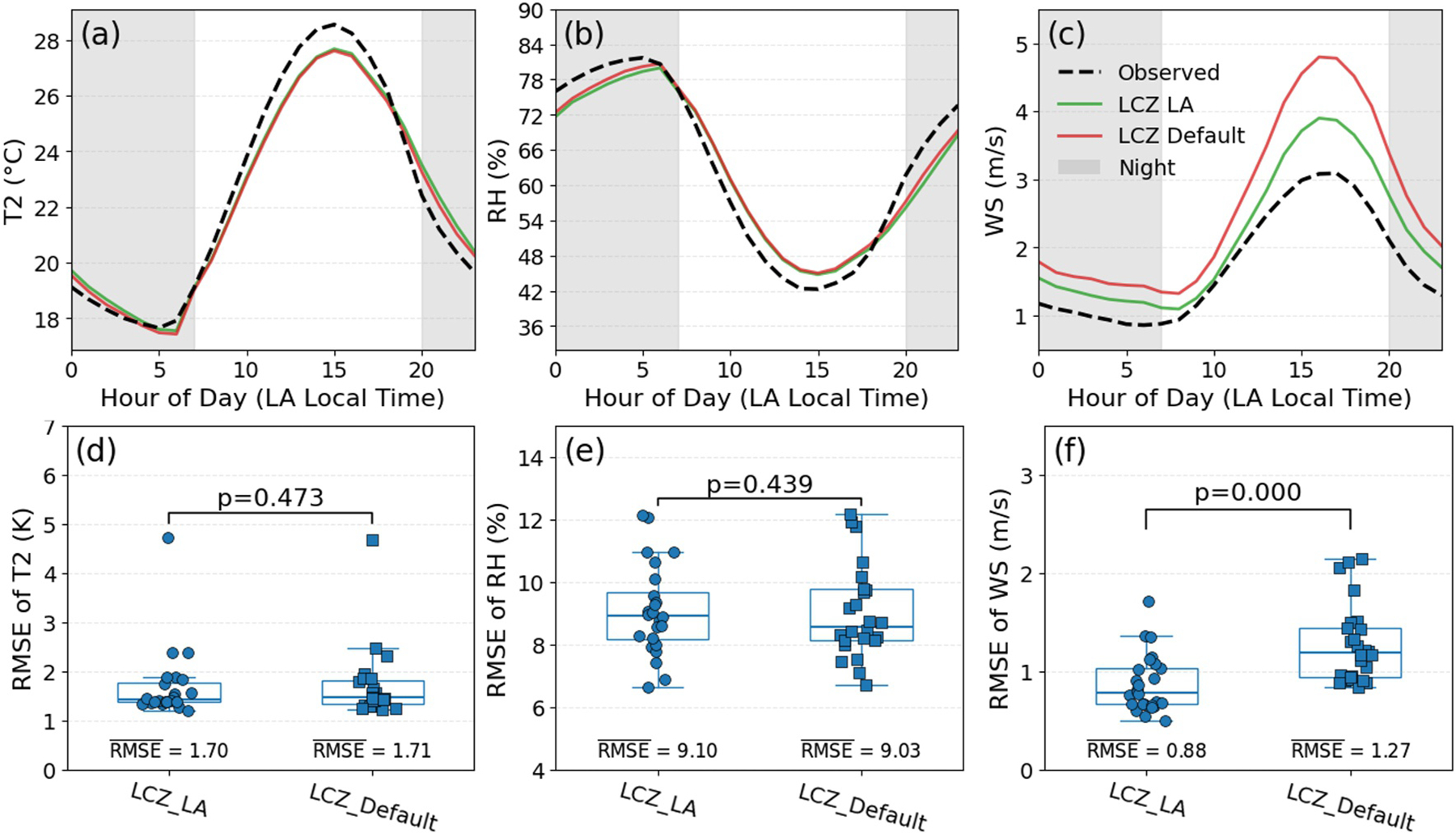
Comparison of model performance under two configurations (LCZ_LA and LCZ_Default). Top panels: diurnal variations of Air Quality System (AQS) site-averaged values for (a) *T*_2_, (b) relative humidity (RH), and (c) wind speed (WS) within the SoCAB. Shaded areas mark nighttime periods (8 p.m.–7 a.m., LA time). Bottom panels: comparison of root mean square error (RMSE) for (d) *T*_2_, (e) RH, and (f) WS at 24 AQS stations. The *p*-values (Wilcoxon test) indicate statistical differences between the RMSE of configurations.

We conducted simulations for the two configurations LCZ_LA and LCZ_Default to estimate the influence of urban UMPs on WRF-SLUCM simulation performance. Diurnal cycles of simulated meteorological variables from two configurations and observed values at the 24 AQS stations are presented in Figures 3a–3c. We utilize RMSE of model simulations relative to hourly AQS observations as the primary evaluation metric to compare configurations and assess the statistical significance of inter-configuration differences using the Wilcoxon test (Figures 3d–3f). Additionally, Figure S2 in Supporting Information S1 provides spatial distributions of the differences between the configurations. On the one hand, adopting LA-customized UMPs increases *T*_2_ and decreases RH over most urban areas in SoCAB, although some coastal areas exhibit reduced *T*_2_ and increased RH. On the other hand, LA-customized UMPs reduce wind speed across all urban grid cells in SoCAB.

For *T*_2_ and RH simulations, we found no significant difference between LCZ_LA and LCZ_Default (Figures 3a, 3b, 3d, and 3e), suggesting that UMPs have limited influence on the evaluation of summertime *T*_2_ and RH simulations in SoCAB. However, as shown in Figure S2 in Supporting Information S1 (a map of average difference between LCZ_LA and LCZ_Default), their difference is substantial in urban areas and can reach 0.6°C for *T*_2_ and 4% for RH. For wind speed simulations, LCZ_LA performs significantly better than LCZ_Default (Figure 3f), which improves wind speed results during both daytime and nighttime (Figure 3c). This highlights the importance of accurate UMPs for urban wind speed simulations. Because the default UMPs are generic rather than LA-specific, a substantial portion of the wind speed bias of LCZ_Default likely arises from a mismatch between the assumed urban morphology and the actual built environment in SoCAB. Our findings are consistent with Shen et al. (2023), who showed that urban morphology has a stronger influence on wind speed than on *T*_2_ and RH.

Although using LA-customized UMPs substantially improves the simulated urban wind fields, overestimation of 10-m wind speed persists over urban areas. Perfect agreement between modeled and observed wind speeds is not expected due to several known factors. First, previous studies suggest that 10-m wind speed output by WRF represents the wind speed at 10 m above the roughness length plus the zero-plane displacement height (Joshi et al., 2025; J. Lee et al., 2022), which is higher than the height of AQS wind speed observation (10 m above the ground). As wind speed typically increases with height, it is likely that the WRF predicted 10-m wind speed would be higher than the observed 10-m wind speed. Second, past WRF simulations have also predicted higher wind speed than observations (He et al., 2019; Sun et al., 2021). This overestimation may be attributed to overly strong turbulent mixing in PBL schemes (D. Chen et al., 2024; X.-M. Hu et al., 2013; Mantovani Júnior et al., 2023; E. Yu et al., 2022) and underestimated effective urban roughness (Cheng et al., 2019; M. Li et al., 2023; Nelli et al., 2020). In this study, we only change UMPs; we use the default urban fraction, aerodynamic roughness length, and all other urban canopy parameters. The refinement of these factors should be considered in future work to further reduce wind speed biases.

### 3.3. Uncertainty Induced by Urban Morphological Parameters

**Figure 4.**
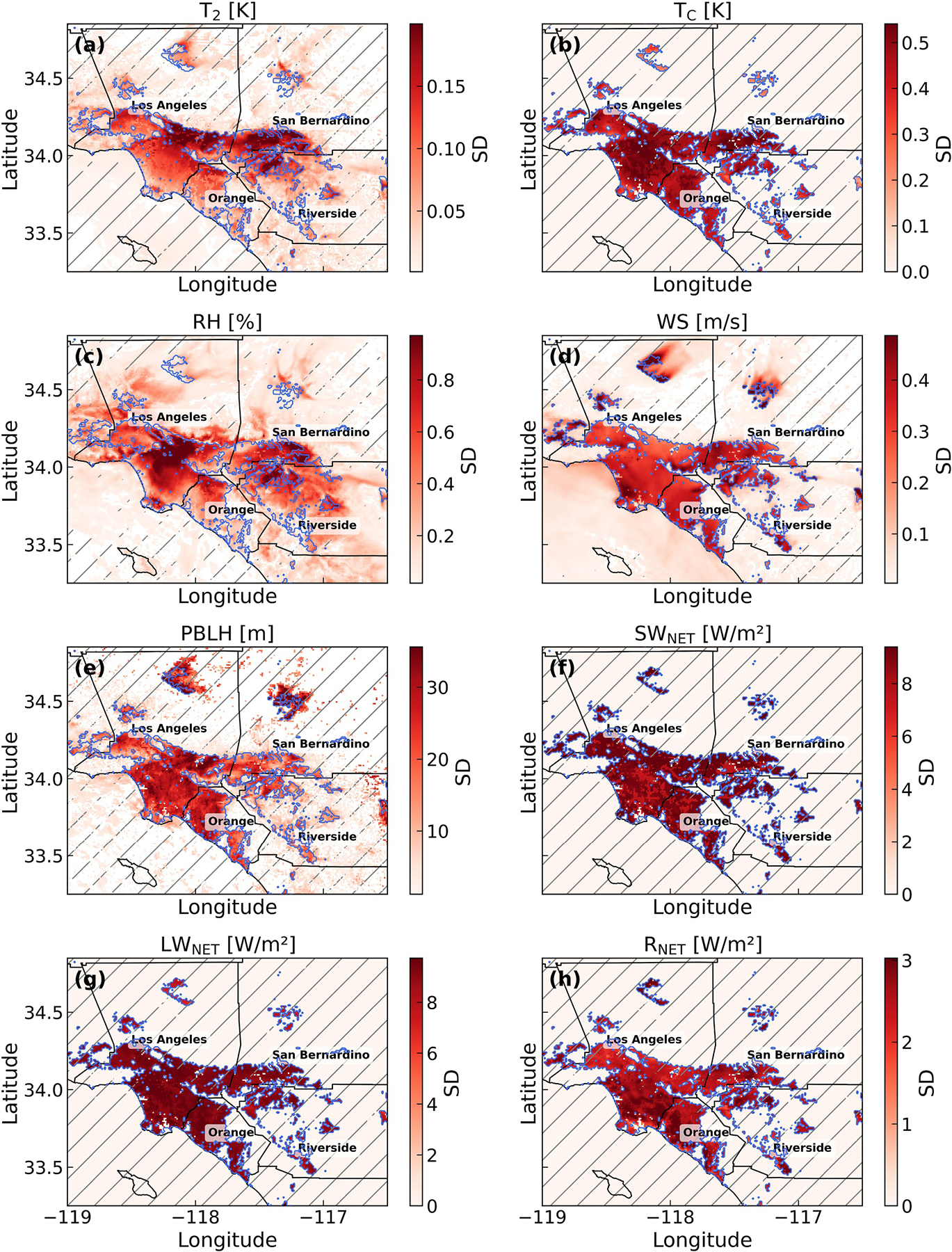
Spatial distribution of uncertainty (SD) in simulated meteorological variables, including (a) *T*_2_, (b) *T*_C_, (c) relative humidity, (d) wind speed, (e) planetary boundary layer height, as well as radiative fluxes, including (f) *SW*_NET_, (g) *LW*_NET_, (h) *R*_NET_, induced by urban morphological parameter perturbations during summer (3 July to 8 July 2019) in the Southern California region. Hatched areas indicate grid cells where the Polynomial Chaos Expansion training *R*^2^ is less than 0.6. Black lines denote county boundaries in Southern California. Blue lines represent the urban boundaries based on the Local Climate Zone land use data.

Figure 4 shows the spatial distribution of uncertainty, represented by the SD of daily-mean WRF-SLUCM outputs, attributable to variations in UMPs across the Southern California region. The estimated uncertainties of all variables (i.e., *T*_2_, *T*_C_, RH, wind speed, PBLH, *SW*_NET_, *LW*_NET_, and *R*_NET_) associated with UMPs are consistently higher in urban areas than in non-urban areas. Most non-urban grid cells have poor PCE fit (*R*^2^ ≤ 0.6) or low uncertainty associated with UMPs (Detailed spatial *R*^2^ map is shown in Figure S3 in Supporting Information S1). This pattern indicates that UMPs primarily influence the simulation results in urban areas, where UMPs directly alter the surface energy balance. Accordingly, our analysis is focused on urban areas in the following sections.

Different variables exhibit distinct spatial patterns of uncertainty. The variation in *T*_C_ shows a similar spatial pattern to the spatial distribution of UMP variations (Figure S4 in Supporting Information S1), as UMPs directly alter the urban energy balance and thus *T*_C_. In contrast, uncertainty in *T*_2_ shows a very different pattern from *T*_C_, with larger values in inland regions. This may be attributed to: (a) the sea breeze transports air from west to east, causing UMP-induced changes to accumulate over inland areas (see the daily average wind vector field in Figure S5 in Supporting Information S1), and (b) inland areas have a lower heat capacity than the ocean and therefore inland areas show a higher response to changes in UMP-induced changes in radiative and heat fluxes.

The uncertainty in *LW*_NET_ and *SW*_NET_ closely follows the spatial patterns of the SDs of the three UMPs (Figure S4 in Supporting Information S1) similar to *T*_C_. Different LCZ types have different UMP variations (Figure S4 in Supporting Information S1), and these UMPs directly control shading, sky-view factor, and multiple reflections between urban surfaces. The uncertainty in *R*_NET_ is smaller than that in *SW*_NET_ and *LW*_NET_ because UMP-induced changes in shortwave and longwave components partially compensate each other.

### 3.4. Diurnal Cycles of Uncertainties

We use PCE to estimate the diurnal cycles of WRF-SLUCM outputs over urban grid cells in summer for *T*_2_, *T*_C_, RH, wind speed, PBLH, *SW*_NET_, *LW*_NET_, and *R*_NET_. The PCE models perform well, as indicated by high cross-validated *R*^2^ (see Figure S6 in Supporting Information S1).

**Figure 5.**
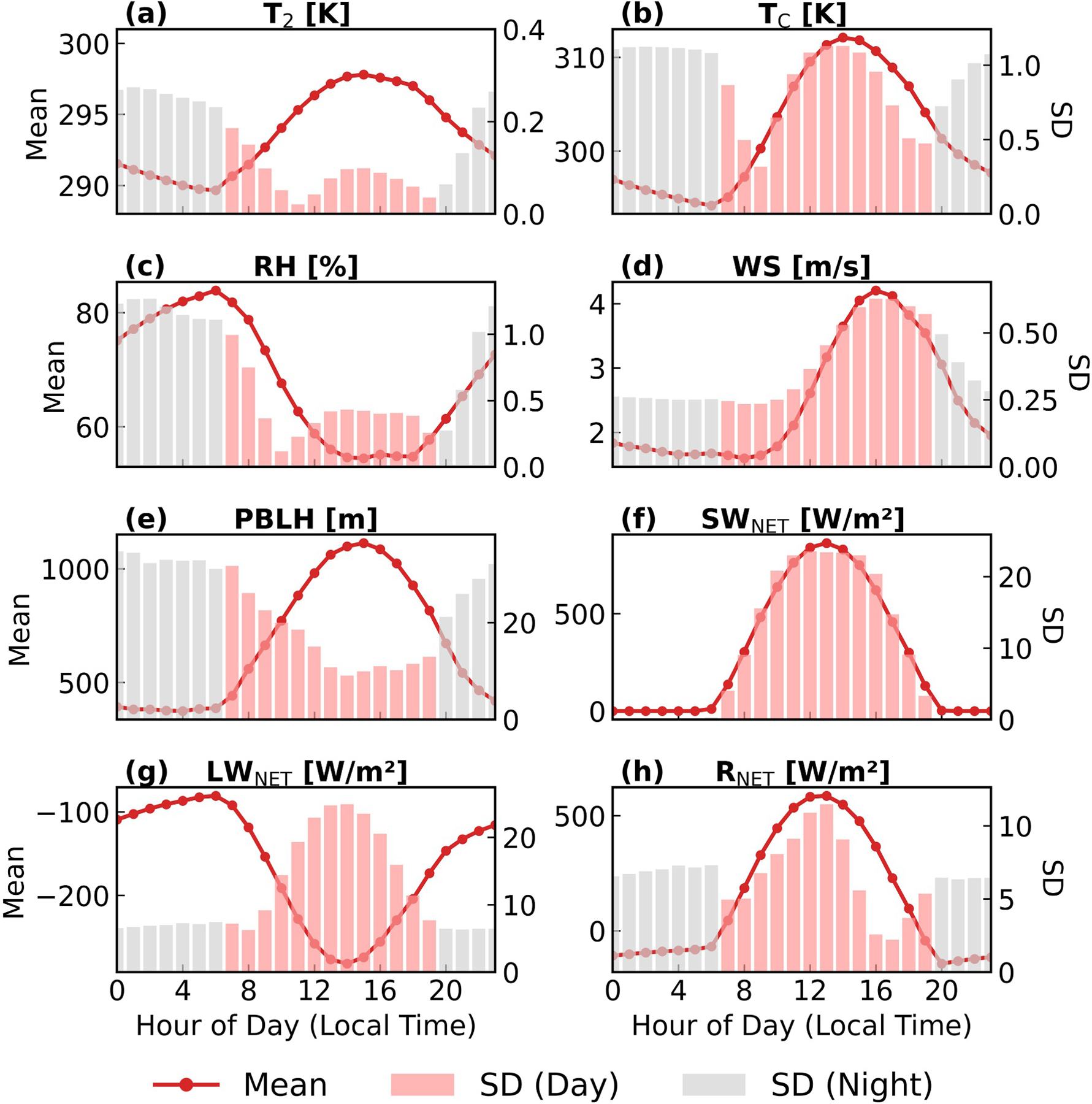
Diurnal variations of Polynomial Chaos Expansion-derived hourly mean and standard deviation (SD) for key WRF-SLUCM outputs in summer over urban areas. The lines represent the hourly mean values, while the bars indicate SD.

Figure 5 presents hourly means and SDs from the PCE surrogate model for these key variables. Nighttime uncertainties are found to be substantially higher than those of daytime, except for wind speed and radiation variables. The underlying reason is that during the day, urban morphology has opposing effects on shortwave and longwave radiations. For example, wider streets increase solar gain but also enhance heat loss due to longwave emissions. The combined impacts of these effects may be smaller. Only processes related to longwave radiation are present at night. This finding is consistent with a previous study by S.-H. Lee et al. (2016), which reported that reducing the aspect ratio from 1.0 to 0.5 led to a greater temperature change at night. Additionally, J. Wang et al. (2023) found that UMPs had a stronger impact on nighttime temperature. Nevertheless, S.-H. Lee et al. (2016) also found that increasing the aspect ratio from 1.0 to 2.0 could result in greater temperature changes during the day. These results imply that the aspect ratio might influence the extent of uncertainty during the day and night. The uncertainty of wind speed follows the diurnal cycle of its absolute value. In urban canopy model, UMPs will influence wind speed via influencing the aerodynamic roughness length (Macdonald et al., 1998), which is calculated including building height, road width and roof width. When the background wind speed is higher, the influence of these roughness-related perturbations becomes greater, resulting in larger wind-speed uncertainty.

From 7 p.m. to around 12 a.m., the SDs of *T*_2_, *T*_C_, RH, and PBLH increase, and then decrease or slightly fluctuate until 7 a.m. During the day, variables respond differently: uncertainty of PBLH reaches its minimum at 2 p.m., while uncertainties of *T*_2_, *T*_C_, and RH reach their minimum at 12 p.m., 10 a.m., and 11 a.m., respectively. For wind speed, the uncertainty reaches its maximum at around 4 p.m. because the uncertainty in wind speed follows its absolute value. Similarly, for radiative fluxes *SW*_NET_ and *LW*_NET_, the diurnal cycles of their uncertainties follow their absolute values, peaking at 12 p.m. and at 2 p.m., respectively. However, for *R*_NET_, which is calculated as *SW*_NET_ plus *LW*_NET_, the uncertainty peaks at 12 p.m., smaller than the uncertainty of *SW*_NET_ and *LW*_NET_.

We further compare the UMP-driven uncertainties with those arising from physics scheme selections (Table 1). On the one hand, uncertainties in *T*_C_, wind speed, and radiation-related variables in urban areas are more sensitive to UMPs than to physics scheme choices, particularly at night. This is likely because UMPs directly influence radiative processes in the urban canopy model, leading to higher uncertainty in urban radiative fluxes, and building height affects surface roughness length, influencing wind speed. On the other hand, the choice of physics schemes exerts a stronger influence on *T*_2_, RH, and PBLH. This may be because these variables represent averages of both urban and non-urban portions of each grid and are more strongly controlled by turbulence, boundary-layer dynamics, and land-atmosphere interactions represented in the physics schemes.

Overall, our results demonstrate that uncertainties in UMPs could lead to significant uncertainty in WRF-SLUCM outputs, underscoring the importance of accurately characterizing UMPs. Such uncertainties may be comparable in magnitude to the impacts of anthropogenic heat and some mitigation strategies assessed by WRF-SLUCM. For example, cool roofs and cool walls have been reported to reduce daily-mean *T*_2_ by about 0.45 and 0.24 K, respectively (Zhang et al., 2019). When applying WRF-SLUCM for policy evaluations, it remains unclear how uncertainties in UMPs might affect these assessments, which highlights the need to investigate potential interactive effects between UMPs and other factors such as anthropogenic heat.

### 3.5. Uncertainty of Urban-Mean Meteorological Variables

**Figure 6.**
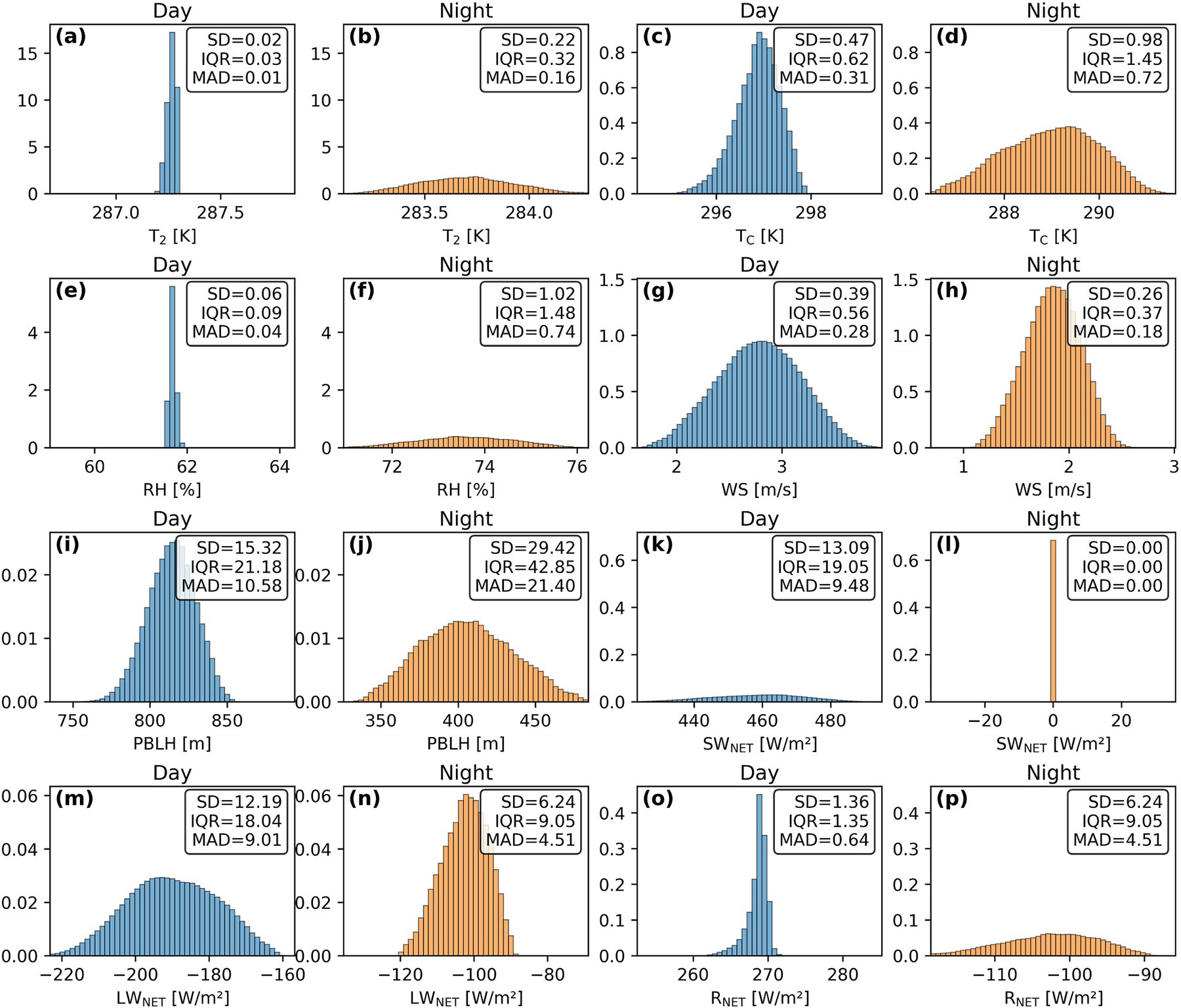
Probability density of output variables (averaged in urban areas in domain 02) from 1,000,000 Monte Carlo simulations varying urban morphological parameters. These variables include *T*_2_ (a, b), *T*_C_ (c, d), relative humidity (e, f), wind speed (g, h), planetary boundary layer height (i, j), *SW*_NET_ (k, l), *LW*_NET_ (m, n), and *R*_NET_ (o, p). Blue bars represent daytime averages, and orange bars represent nighttime averages.

We perform PCE fitting on daytime and nighttime meteorological variables and energy fluxes averaged over urban grid cells in Southern California and found that the trained model produced high *R*^2^ values for all variables (see Figure S7 in Supporting Information S1 for more details). We then used the PCE surrogate model combined with the Monte Carlo approach to generate 1,000,000 simulations of daytime and nighttime urban averages, enabling further uncertainty and output distribution analysis shown in Figure 6.

Figure 6 indicates that, despite the UMPs being drawn from a uniform distribution, the outputs of the WRF-SLUCM simulations, such as temperature and wind speed, approximately display a near-Gaussian distribution. This may be due to the independence of the three UMPs and the limited role of their interactions (discussed in Section 3.6), which, when combined through the model’s nonlinear processes, produces output distributions with higher density near central values. We further investigate the effects of individual UMPs, as shown in Figures S8–S10 in Supporting Information S1, and find that the output variables’ distributions are skewed or U-shaped, which is related to the nonlinearity and monotonicity in the WRF-SLUCM parameterizations.

### 3.6. Sensitivity Analysis of Individual Urban Morphological Parameters

We apply the PCE method and Sobol global sensitivity analysis to quantify the first- and second-order effects of each UMP, including building height (H), road width (W), and roof width (R), on WRF-SLUCM model outputs.

**Figure 7.**
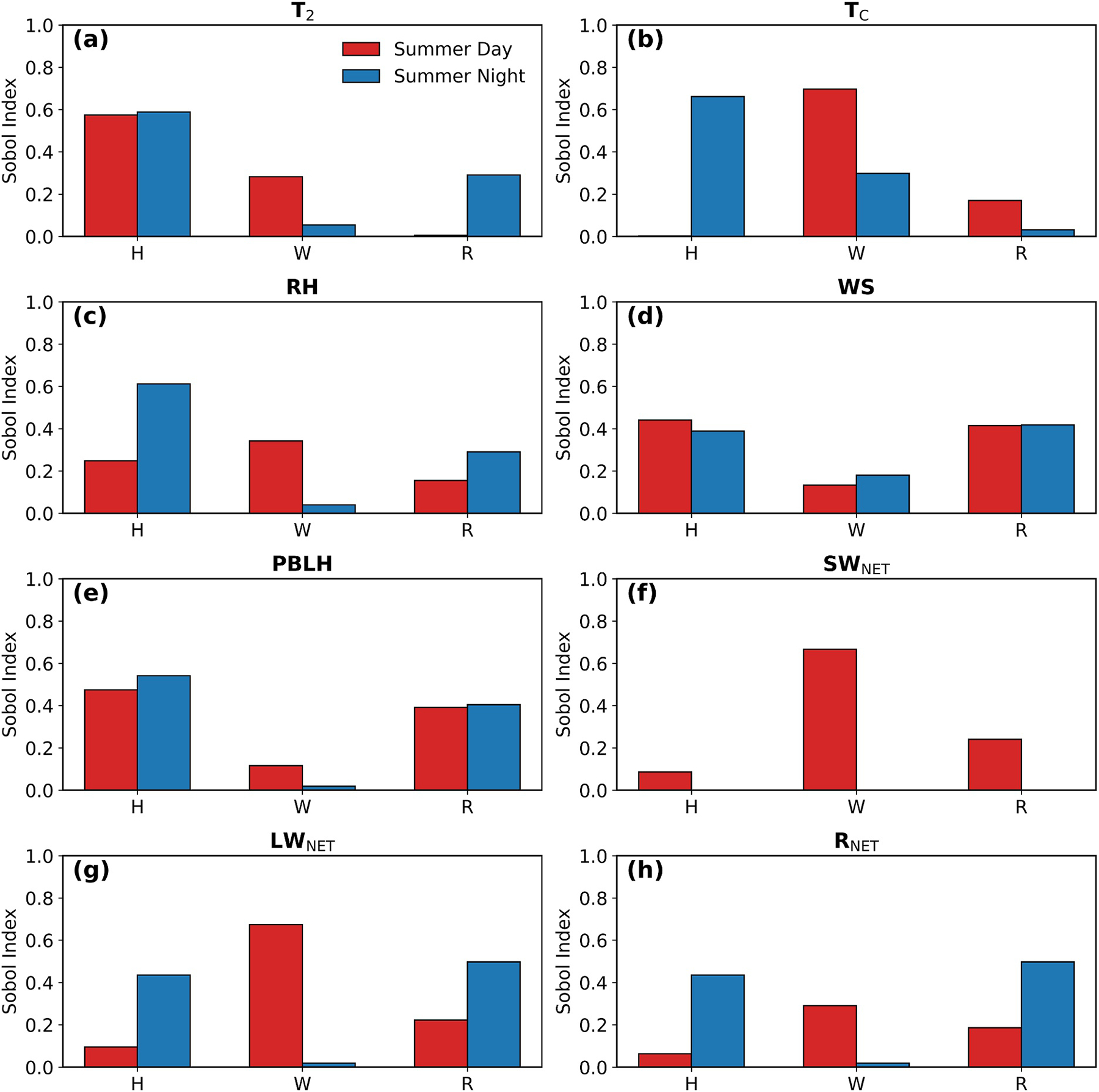
Sobol index of urban morphology parameters, including building height (H), road width (W), and roof width (R) for all WRF-SLUCM output variables (*T*_2_, *T*_C_, relative humidity, wind speed, planetary boundary layer height, *SW*_NET_, *LW*_NET_, and *R*_NET_), averaged over urban areas.

Figure 7 shows the relative importance of individual parameters in simulating meteorological variables and radiative fluxes. Variations in meteorological variables within WRF–SLUCM are driven primarily by the direct effects of UMPs (first-order Sobol index). The interaction terms, represented by the second-order Sobol indices, can be interpreted as indicating whether the effect of one parameter on the output depends on the value of another parameter. As shown in Figure S11 in Supporting Information S1, interaction effects (second-order Sobol index) are generally minor, indicating that uncertainties in urban climate simulations in this study arise mainly from individual parameter contributions rather than inter-parameter interactions.

Meteorological variables and radiative fluxes have different controlling UMPs. Urban-mean *T*_2_ (Figure 7a) is most sensitive to building height; the sensitivity to road width exceeds that to roof width during daytime, but the pattern reverses at night. Wind speed is consistently driven by building height and roof width, as shown in Figure 7d. Unlike previous studies that focused only on meteorological variables and the canyon aspect ratio (the ratio of building height to road width) (Hang & Chen, 2022; S.-H. Lee et al., 2016; Theeuwes et al., 2014), we adopt a statistical perspective to report the relative importance of each individual parameter on the model outputs, which is calculated based on the input variations, as well as their interaction effects, on the model outputs.

We note as a caveat that all UMPs are standardized to their baseline LA-customized values and perturbed by ±50%, so the results capture only the sensitivity of meteorological outputs under our particular baseline values. They do not reveal the direction of responses (i.e., whether a UMP increases or decreases a given output) or quantify the marginal effects associated with incremental parameter changes, both of which have been addressed in previous sensitivity analyses. At the same time, the spatial heterogeneity of this sensitivity remains to be investigated. Despite these limitations, our results show the relative importance of UMPs in influencing urban climate variables.

### 3.7. Uncertainty and Sensitivity Analysis of Heat Stress Indicators

**Figure 8.**
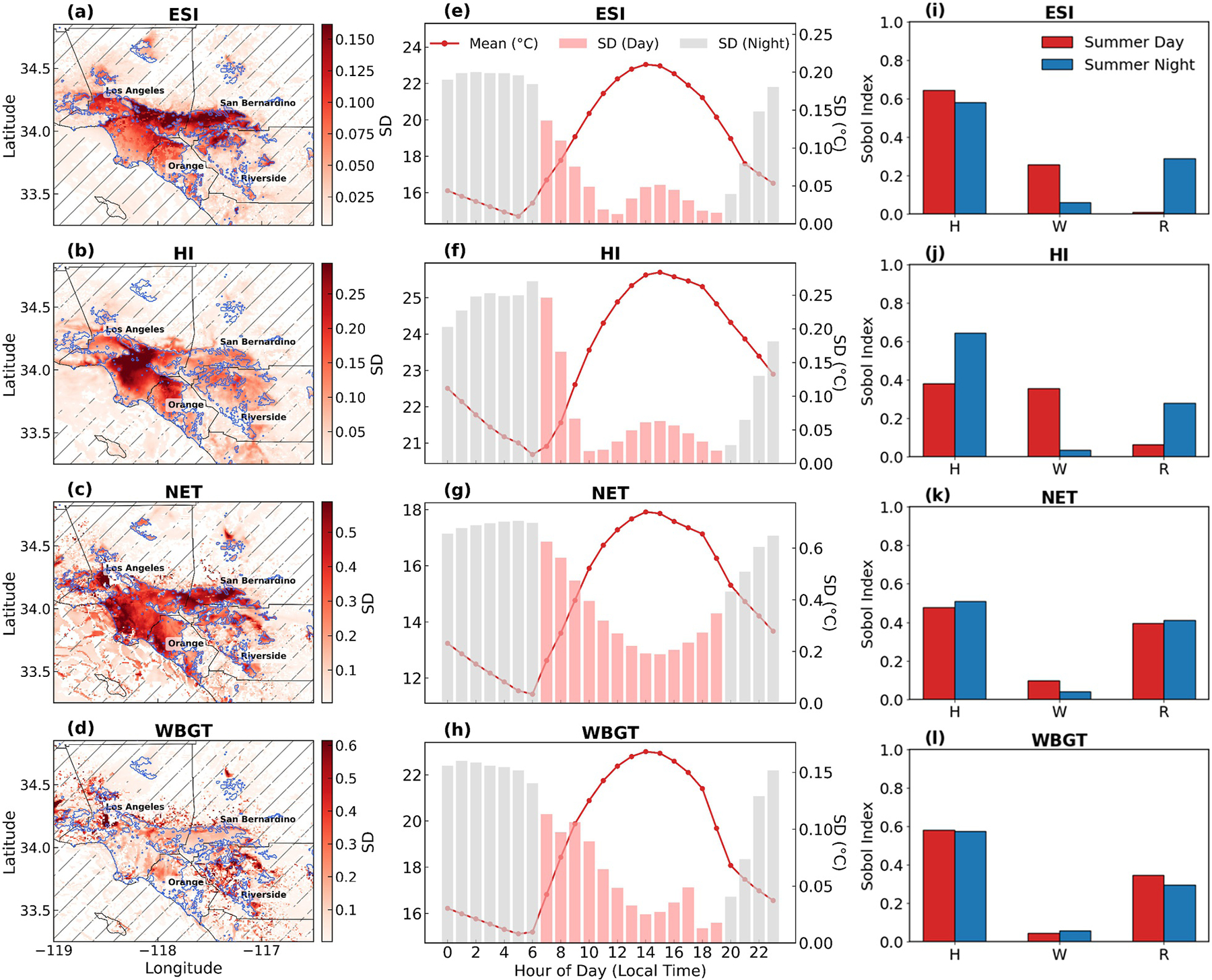
Uncertainty and sensitivity analysis of four heat stress indicators under varying urban morphological parameters in the Los Angeles region. (a–d): Maps of standard deviations for daily average (a) Environmental Stress Index (ESI), (b) heat index (HI), (c) Net Effective Temperature (NET), and (d) Wet Bulb Globe Temperature (WBGT). Hatched areas indicate regions excluded from the analysis due to the poor performance (*R*^2^ > 0.6). Black lines denote county boundaries in Southern California. Blue lines represent the urban boundaries based on Local Climate Zone land use data. (e–h): Diurnal cycles and standard deviations (pink bars, right axis) for (e) ESI, (f) HI, (g) NET, and (h) WBGT, indicating temporal patterns of uncertainty. (i–l): Sobol sensitivity indices for (i) ESI, (j) HI, (k) NET, and (l) WBGT under summer daytime (red) and nighttime (blue) conditions.

For the first time, we quantitatively assess the contribution of variations in UMPs to uncertainties in heat stress indices (ESI, HI, NET, and WBGT) in Southern California. The spatial distributions of uncertainties (Figures 8a–8d), temporal patterns of uncertainties (Figures 8e–8h), and Sobol sensitivity indices (Figures 8i–8l). Additional details on the performance of the PCE model, including *R*^2^ values for urban and non-urban areas and their hourly variations, are provided in Figures S12 and S13 in Supporting Information S1.

The influence of UMPs on heat stress propagates along the computational chain from meteorological variables to the derived indices. Figures 8a–8d suggest that the four heat-stress indices exhibit distinct spatial patterns of UMP-induced uncertainty, whereas Figures 8e–8h indicate a similar diurnal pattern, with lower uncertainty during the day and higher at night. Among the indicators, ESI exhibits spatial and temporal uncertainty patterns that closely mirror those of *T*_2_ (see Figure 4), while HI shows stronger alignment with RH. In both cases, building height is the dominant contributor to uncertainty, as reflected in the Sobol index values (Figures 8i and 8j). In contrast, NET and WBGT demonstrate more complex uncertainty characteristics due to their more intricate formulations, which involve nonlinear or empirical components beyond simple arithmetic expressions. Temporally, both indicators exhibit lower uncertainty during the daytime than at night (Figures 8g and 8h). Sensitivity analysis further reveals that NET and WBGT are influenced most significantly by building height and roof width (Figures 8k–8l), a pattern distinct from ESI and HI, underscoring the importance of multiple morphological parameters in shaping uncertainty for more complex heat stress indices. Furthermore, all four indicators exhibit higher uncertainty in urban areas and during nighttime periods relative to non-urban areas and daytime, respectively.

Our findings indicate that different heat stress indicators exhibit distinct uncertainty patterns in response to variations in UMPs, reflecting differences in their calculation approaches and the meteorological variables involved. This highlights the importance of accurately representing UMPs in models, as they influence heat stress indicators and can otherwise bias subsequent heat-related health assessments.

## 4. Conclusions

This study provides the first comprehensive evaluation of how land-use data and UMPs affect WRF-SLUCM performance in Southern California. We developed a framework that integrates PCE, Sobol sensitivity analysis, and Monte Carlo methods to quantify uncertainties in WRF-SLUCM simulations arising from variations in UMPs. To our knowledge, this is the first application of PCE to evaluate the uncertainty arising from UMP variations within the fully coupled WRF-SLUCM model. Additionally, we present the first assessment of how UMP-related uncertainties influence heat stress indicators in the region.

We find that applying LA-customized UMPs in LCZ land use data has a negligible effect on *T*_2_ or RH simulations in Southern California but significantly improves wind speed simulations. Next, we conduct a series of spatial and temporal uncertainty analyses. The uncertainty analysis reveals a strong sensitivity of urban meteorological conditions to a 50% perturbation in UMPs. For example, the resulting average SDs of 2 m air temperature are 0.02 K during the day and 0.23 K at night, whereas those of urban canyon temperature reach 0.51 and 1.03 K, respectively. Uncertainties in *T*_C_, wind speed, and radiative fluxes caused by varying UMPs exceed those from physics scheme selections, highlighting the critical role of morphology in urban climate modeling. These uncertainties are mainly concentrated in urban areas and display similar diurnal patterns, remaining higher at night but becoming lower during the day due to the complex offsetting effects from shortwave and longwave radiations.

Sobol sensitivity analysis indicates that the effects of UMPs are primarily driven by their direct contributions, with interaction effects being negligible. Meteorological variables and radiative fluxes have different controlling UMPs. Building height and road width have been extensively studied in previous research. However, roof width —often overlooked in previous analyses that emphasized aspect ratios—also plays an important role in influencing WRF–SLUCM outputs.

Furthermore, we find that different heat stress indicators (ESI, HI, NET, WBGT) show different uncertainty patterns induced by UMP variations, which is attributed to their variant calculation methods and dependent meteorological variables. For example, NET shows the largest among the four indicators, up to 0.6 K at night, owing to the propagation of wind speed’s large uncertainty, while ESI and HI exhibit uncertainty patterns that are largely similar to those of *T*_2_ and RH.

We acknowledge that other factors such as different seasons and the presence or absence of heatwave conditions may also lead to distinct uncertainties induced by UMPs. Additionally, understanding how these uncertainties propagate to air-quality predictions is an important direction for future research. Our uncertainty quantification framework can be extended to investigate these research questions. We also suggest further research to examine the physical mechanisms driving UMP-related uncertainties, including their spatial patterns.

Overall, these findings provide new insights for urban climate modelers regarding the impacts of land use data and urban morphology parameterization, while presenting a useful framework for uncertainty quantification. By identifying and characterizing these uncertainties, modelers can track error sources, improve model inputs, and avoid misleading conclusions, which have significant implications for future urban heat-induced health assessments.

## Supplementary Material

Supporting information S1

Supporting Information may be found in the online version of this article.

## Figures and Tables

**Table 1:** Comparison of Uncertainties in WRF-SLUCM Output Variables Averaged in Urban Areas (Expressed in Terms of Standard Deviations) Induced by Urban Morphology and Physical Parameterizations

Standard deviation	Induced by urban morphology		Induced by physics schemes	
Variables	Day	Night	Day	Night
*T*_2_ (K)	0.02	0.23	0.38	0.40
*T*_C_ (K)	0.51	1.03	0.22	0.16
RH (%)	0.07	1.08	2.24	4.29
Wind speed (m/s)	0.42	0.27	0.29	0.13
PBLH (m)	16.25	31.20	169.78	109.74
*SW*_NET_ (W/m^2^)	13.79	–	2.63	–
*LW*_NET_ (W/m^2^)	12.81	6.63	1.44	1.21
*R*_NET_ (W/m^2^)	1.70	6.63	2.56	1.21

## Data Availability

The source code of the Weather Research and Forecasting (WRF) Model Version 4.6.1 coupled with the single-layer urban canopy model (SLUCM), along with all required model files, is publicly available from the official WRF website https://github.com/wrf-model/WRF/tree/release-v4.6.1 (NCAR/UCAR, 2024). The modified WRF-SLUCM source files used in this study are archived at https://github.com/ACE-lab-USC/WRF-SLUCM-Modified (H. Hu, 2026a). Ground-based air quality data were obtained from the U.S. Environmental Protection Agency’s Air Quality System (AQS) database (US EPA, 2012). The core implementation of the PCE-based uncertainty quantification framework, together with a minimal reproducible example, is preserved at Zenodo (H. Hu, 2026b). To limit file size, the shared input data set for this example consists of urban- and non-urban-mean values of the WRF output variables from the 100-member ensemble used to train the PCE surrogate. Additional input and output data from the full set of WRF simulations, as well as other supporting data sets, are available from the corresponding author upon request.

## Key Points:

- Using local climate zone (LCZ) customized urban morphological parameters (UMPs) improves wind speed simulations significantly in Southern California
- Urban wind and canyon temperature are more sensitive to building height, road, and roof width than to model physics scheme choice
- Input UMP uncertainties lead to varying uncertainty levels across estimated urban heat stress indices

## References

[R1] Affairs U. N. D. E. S. (2019). World Urbanization prospects: The 2018 revision. United Nations. 10.18356/b9e995fe-en

[R2] Berg LK, Gustafson WI, Kassianov EI, & Deng L (2013). Evaluation of a modified scheme for shallow convection: Implementation of CuP and case studies. Monthly Weather Review, 141(1), 134–147. 10.1175/MWR-D-12-00136.1

[R3] Budd GM (2008). Wet-bulb globe temperature (WBGT)—Its history and its limitations. Journal of Science and Medicine in Sport, 11(1), 20–32. 10.1016/j.jsams.2007.07.003 17765661

[R4] Buzan JR, Oleson K, & Huber M (2015). Implementation and comparison of a suite of heat stress metrics within the Community Land Model version 4.5. Geoscientific Model Development, 8(2), 151–170. 10.5194/gmd-8-151-2015

[R5] Chen B, Wang W, Dai W, Chang M, Wang X, You Y, (2021). Refined urban canopy parameters and their impacts on simulation of urbanization-induced climate change. Urban Climate, 37, 100847. 10.1016/j.uclim.2021.100847

[R6] Chen D, Zhu A, Huang L, Yaluk E, Wang Y, Ooi MCG, (2024). Sensitivity analysis of planetary boundary layer parameterization on meteorological simulations in the Yangtze river delta region. China, 4(10), 1129–1144. 10.1039/D4EA00038B

[R7] Chen F, Kusaka H, Bornstein R, Ching J, Grimmond CSB, Grossman-Clarke S, (2011). The integrated WRF/urban modelling system: Development, evaluation, and applications to urban environmental problems. International Journal of Climatology, 31(2), 273–288. 10.1002/joc.2158

[R8] Chen F, Yang X, & Zhu W (2014). WRF simulations of urban heat island under hot-weather synoptic conditions: The case study of Hangzhou City, China. Atmospheric Research, 138, 364–377. 10.1016/j.atmosres.2013.12.005

[R9] Chen G, Zhao L, & Mochida A (2016). Urban heat Island simulations in Guangzhou, China, using the coupled WRF/UCM model with a land use map extracted from remote sensing data. Sustainability, 8(7), 628. 10.3390/su8070628

[R10] Cheng F-Y, Lin C-F, Wang Y-T, Tsai J-L, Tsuang B-J, & Lin C-H (2019). Impact of effective roughness length on mesoscale meteorological simulations over heterogeneous land surfaces in Taiwan. Atmosphere, 10(12), 805. 10.3390/atmos10120805

[R11] Ching J, Brown M, Burian S, Chen F, Cionco R, Hanna A, (2009). National urban database and access portal tool. Bulletin of the American Meteorological Society, 90(8), 1157–1168. 10.1175/2009BAMS2675.1

[R12] Commerce NCEPWSSD. (2015). NCEP GDAS/FNL 0.25 degree global tropospheric analyses and forecast grids [Dataset]. UCAR/NCAR Research Data Archive. 10.5065/D65Q4T4Z

[R13] County of Los Angeles. (2014). LARIAC4 buildings 2014, County of Los Angeles open data portal [Dataset]. Retrieved from https://data.lacounty.gov/datasets/lariac4-buildings-2014/about

[R14] Courant R, Friedrichs K, & Lewy H (1928). Über die partiellen Differenzengleichungen der mathematischen Physik. Mathematische Annalen, 100(1), 32–74. 10.1007/BF01448839

[R15] Demuzere M, He C, Martilli A, & Zonato A (2023). Technical documentation for the hybrid 100-m global land cover dataset with Local Climate zones for WRF. 10.5281/zenodo.7670791

[R16] Ding W, & Chen H (2024). Investigating the microclimate impacts of blue–green space development in the urban–rural fringe using the WRF-UCM model. Urban Climate, 54, 101865. 10.1016/j.uclim.2024.101865

[R17] Du Y, Xu T, Che Y, Yang B, Chen S, Su Z, (2022). Uncertainty quantification of WRF model for rainfall prediction over the Sichuan Basin, China. Atmosphere, 13(5), 838. 10.3390/atmos13050838

[R18] Emery C, Liu Z, Russell AG, Odman MT, Yarwood G, & Kumar N (2017). Recommendations on statistics and benchmarks to assess photochemical model performance. Journal of the Air & Waste Management Association, 67(5), 582–598. 10.1080/10962247.2016.1265027 27960634

[R19] Epstein SA, Lee S-M, Katzenstein AS, Carreras-Sospedra M, Zhang X, Farina SC, (2017). Air-quality implications of wide-spread adoption of cool roofs on ozone and particulate matter in southern California. Proceedings of the National Academy of Sciences, 114(34), 8991–8996. 10.1073/pnas.1703560114 PMC5576793 28784778

[R20] Gutiérrez E, González JE, Martilli A, Bornstein R, & Arend M (2015). Simulations of a heat-wave event in New York City using a multilayer urban parameterization. 10.1175/JAMC-D-14-0028.1

[R21] Hang J, & Chen G (2022). Experimental study of urban microclimate on scaled street canyons with various aspect ratios. Urban Climate, 46, 101299. 10.1016/j.uclim.2022.101299

[R22] He X, Li Y, Wang X, Chen L, Yu B, Zhang Y, & Miao S (2019). High-resolution dataset of urban canopy parameters for Beijing and its application to the integrated WRF/Urban modelling system. Journal of Cleaner Production, 208, 373–383. 10.1016/j.jclepro.2018.10.086

[R23] Heaviside C, Macintyre H, & Vardoulakis S (2017). The urban heat Island: Implications for health in a changing environment. Current Environmental Health Reports, 4(3), 296–305. 10.1007/s40572-017-0150-3 28695487

[R24] Herrmann M, & Gutheil E (2022). Simulation of the Air Quality in Southern California, USA in July and October of the Year 2018. Atmosphere, 13(4), 548. 10.3390/atmos13040548

[R25] Homer C, Dewitz J, Yang L, Jin S, Danielson P, Xian G, (2015). Completion of the 2011 national land cover database for the conterminous United States—Representing a decade of land cover change information. Photogrammetric Engineering & Remote Sensing, 81(5), 345–354. 10.1016/S0099-1112(15)30100-2

[R26] Hu H (2026a). ACE-lab-USC/WRF-SLUCM-Modified [Software]. GitHub. Retrieved from https://github.com/ACE-lab-USC/WRF-SLUCM-Modified

[R27] Hu H (2026b). Quantifying urban morphology-induced uncertainty in urban meteorology and heat stress simulations in Southern California [Dataset]. Zenodo. 10.5281/zenodo.18510288 PMC13089901 42007180

[R28] Hu X-M, Klein PM, & Xue M (2013). Evaluation of the updated YSU planetary boundary layer scheme within WRF for wind resource and air quality assessments. Journal of Geophysical Research: Atmospheres, 118(18), 10490–10505. 10.1002/jgrd.50823

[R29] Hulley GC, Dousset B, & Kahn BH (2020). Rising trends in heatwave metrics across Southern California. Earth’s Future, 8(7), e2020EF001480. 10.1029/2020EF001480

[R30] Iacono MJ, Delamere JS, Mlawer EJ, Shephard MW, Clough SA, & Collins WD (2008). Radiative forcing by long-lived greenhouse gases: Calculations with the AER radiative transfer models. Journal of Geophysical Research, 113(D13). 10.1029/2008JD009944

[R31] Jin J, Che Y, Zheng J, & Xiao F (2019). Uncertainty quantification of a coupled model for wind prediction at a wind farm in Japan. Energies, 12(8), 1505. 10.3390/en12081505

[R32] Joshi P, Lin T-S, He C, & Lamer K (2025). Urban weather modeling using WRF: Linking physical assumptions, code implementation, and observational needs. Geoscientific Model Development, 18(20), 7869–7890. 10.5194/gmd-18-7869-2025

[R33] Kotharkar R, Dongarsane P, & Keskar R (2023). Determining influence of urban morphology on air temperature and heat index with hourly emphasis. Building and Environment, 233, 110044. 10.1016/j.buildenv.2023.110044

[R34] Kumar M, Kosović B, Nayak HP, Porter WC, Randerson JT, & Banerjee T (2024). Evaluating the performance of WRF in simulating winds and surface meteorology during a Southern California wildfire event. Frontiers in Earth Science, 11, 1305124. 10.3389/feart.2023.1305124

[R35] Kusaka H, Kondo H, Kikegawa Y, & Kimura F (2001). A simple single-layer urban canopy model for atmospheric models: Comparison with multi-layer and slab models. Boundary-Layer Meteorology, 101(3), 329–358. 10.1023/A:1019207923078

[R36] Lee J, Lee H-J, Kim K-B, Shin HH, Lim J-M, Hong J, & Lim K-SS (2022). Height correction method based on the Monin–Obukhov similarity theory for better prediction of near-surface wind fields. In SSRN scholarly paper, Rochester, NY. Social Science Research Network. 10.2139/ssrn.4133432

[R37] Lee S-H, Lee H, Park S-B, Woo J-W, Lee D-I, & Baik J-J (2016). Impacts of in-canyon vegetation and canyon aspect ratio on the thermal environment of street canyons: Numerical investigation using a coupled WRF-VUCM model. Quarterly Journal of the Royal Meteorological Society, 142(699), 2562–2578. 10.1002/qj.2847

[R38] Lemonsu A, Grimmond CSB, & Masson V (2004). Modeling the surface energy balance of the core of an old Mediterranean City: Marseille. Retrieved from https://journals.ametsoc.org/view/journals/apme/43/2/1520-0450_2004_043_0312_mtsebo_2.0.co_2.xml

[R39] Li D, Sun T, Yang J, Zhang N, Vahmani P, & Jones A (2024). Structural uncertainty in the sensitivity of urban temperatures to anthropogenic heat flux. Journal of Advances in Modeling Earth Systems, 16(10), e2024MS004431. 10.1029/2024MS004431

[R40] Li M, Zhang JA, Matak L, & Momen M (2023). The impacts of adjusting momentum roughness length on strong and weak hurricane forecasts: A comprehensive analysis of weather simulations and observations. Monthly Weather Review, 151(5), 1287–1302. 10.1175/MWR-D-22-0191.1

[R41] Li PW, & Chan ST (2000). Application of a weather stress index for alerting the public to stressful weather in Hong Kong. Meteorological Applications, 7(4), 369–375. 10.1017/S1350482700001602

[R42] Li Y, Zhang J, Sailor DJ, & Ban-Weiss GA (2019). Effects of urbanization on regional meteorology and air quality in Southern California. Atmospheric Chemistry and Physics, 19(7), 4439–4457. 10.5194/acp-19-4439-2019

[R43] Liao W, Li Y, Liu X, Wang Y, Che Y, Shao L, (2024). GloUCP: A global 1 km spatially continuous urban canopy parameters for the WRF model. ESSD Land/Land Cover and Land Use. 10.5194/essd-2024-408

[R44] Liu Y, Xu Y, Zhang F, & Shu W (2020). A preliminary study on the influence of Beijing urban spatial morphology on near-surface wind speed. Urban Climate, 34, 100703. 10.1016/j.uclim.2020.100703

[R45] Macdonald RW, Griffiths RF, & Hall DJ (1998). An improved method for the estimation of surface roughness of obstacle arrays. Atmospheric Environment, 32(11), 1857–1864. 10.1016/S1352-2310(97)00403-2

[R46] Mantovani Júnior JA, Aravéquia JA, Carneiro RG, & Fisch G (2023). Evaluation of PBL parameterization schemes in WRF model predictions during the dry season of the central Amazon Basin. Atmosphere, 14(5), 850. 10.3390/atmos14050850

[R47] Masson V (2000). A physically-based scheme for the urban energy budget in atmospheric models. Boundary-Layer Meteorology, 94(3), 357–397. 10.1023/A:1002463829265

[R48] Moosavi A, Rao V, & Sandu A (2021). Machine learning based algorithms for uncertainty quantification in numerical weather prediction models. Journal of Computational Science, 50, 101295. 10.1016/j.jocs.2020.101295

[R49] Moran DS, & Epstein Y (2006). Evaluation of the Environmental Stress Index (ESI) for Hot/Dry and Hot/Wet climates. Industrial Health, 44(3), 399–403. 10.2486/indhealth.44.399 16922183

[R50] Morrison H, Thompson G, & Tatarskii V (2009). Impact of cloud microphysics on the development of trailing stratiform precipitation in a simulated squall line: Comparison of One- and two-moment schemes. Monthly Weather Review, 137(3), 991–1007. 10.1175/2008MWR2556.1

[R51] NCAR/UCAR. (2024). WRF version 4.6.1 [Software]. GitHub. Retrieved from https://github.com/wrf-model/WRF/tree/release-v4.6.1

[R52] Nelli NR, Temimi M, Fonseca RM, Weston MJ, Thota MS, Valappil VK, (2020). Impact of roughness length on WRF simulated land-atmosphere interactions over a hyper-arid Region. Earth and Space Science, 7(6), e2020EA001165. 10.1029/2020EA001165

[R53] Palar PS, Zuhal LR, Shimoyama K, & Tsuchiya T (2018). Global sensitivity analysis via multi-fidelity polynomial chaos expansion. Reliability Engineering & System Safety, 170, 175–190. 10.1016/j.ress.2017.10.013

[R54] Qian J, Zhang L, Schlink U, Hu X, Meng Q, & Gao J (2025). Impact of urban land use and anthropogenic heat on winter and summer outdoor thermal comfort in Beijing. Urban Climate, 59, 102306. 10.1016/j.uclim.2025.102306

[R55] Qin Y, Liao W, & Li D (2023). Attributing the urban–rural contrast of heat stress simulated by a global model. Journal of Climate, 36(6), 1805–1822. 10.1175/JCLI-D-22-0436.1

[R56] Schlaerth HL, Silva SJ, Li Y, & Li D (2023). Albedo as a competing warming effect of urban greening. Journal of Geophysical Research: Atmospheres, 128(24), e2023JD038764. 10.1029/2023JD038764

[R57] Shen C, Liu Y, Dai W, Chen X, Fan Q, Wang X, (2023). The influence of refined urban morphological parameters on dynamical and thermal fields in a single-layer urban canopy model. Atmosphere, 14(4), 719. 10.3390/atmos14040719

[R58] Skamarock WC, Klemp JB, Dudhia J, Gill DO, Liu Z, Berner J, (2019). A description of the advanced research WRF model version 4.

[R59] Smithson C, & Adams BR (2024). Use of LCZs with urban canopy modeling to evaluate urban growth effects on meteorological conditions in the Salt Lake Valley. Urban Climate, 57, 102106. 10.1016/j.uclim.2024.102106

[R60] South Coast AQMD. (2022). South Coast AQMD air quality management plan. Retrieved from https://www.scaqmd.gov/home/air-quality/air-quality-management-plans/air-quality-mgt-plan/2022-aqmp-archive

[R61] Stewart ID, & Oke TR (2012). Local climate zones for urban temperature studies. Bulletin of the American Meteorological Society, 93(12), 1879–1900. 10.1175/BAMS-D-11-00019.1

[R62] Stull R (2011). Wet-Bulb temperature from relative humidity and air temperature. Journal of Applied Meteorology and Climatology, 50(11), 2267–2269. 10.1175/JAMC-D-11-0143.1

[R63] Sukoriansky S, Galperin B, & Perov V (2005). Application of a new spectral theory of stably stratified turbulence to the atmospheric boundary layer over Sea ice. Boundary-Layer Meteorology, 117(2), 231–257. 10.1007/s10546-004-6848-4

[R64] Sun Y, Zhang N, Miao S, Kong F, Zhang Y, & Li N (2021). Urban morphological parameters of the main cities in China and their application in the WRF model. Journal of Advances in Modeling Earth Systems, 13(8), e2020MS002382. 10.1029/2020MS002382

[R65] Tam BY, Gough WA, & Mohsin T (2015). The impact of urbanization and the urban heat island effect on day to day temperature variation. Urban Climate, 12, 1–10. 10.1016/j.uclim.2014.12.004

[R66] Tewari M (2004). Implementation and verification of the unified Noah land surface model in the WRF model. Retrieved from https://cir.nii.ac.jp/crid/1370298336568217475

[R67] Theeuwes NE, Steeneveld GJ, Ronda RJ, Heusinkveld BG, van Hove LWA, & Holtslag AAM (2014). Seasonal dependence of the urban heat island on the street canyon aspect ratio. Quarterly Journal of the Royal Meteorological Society, 140(684), 2197–2210. 10.1002/qj.2289

[R68] US Census Bureau, USCB. (2022). U.S. census bureau. Retrieved from https://www.census.gov/quickfacts/fact/table/losangelescountycalifornia,CA/PST045222

[R69] US EPA. (2012). Air quality System data Mart [Dataset]. Data & Tools. Retrieved from https://aqs.epa.gov/aqsweb/airdata/download_files.html

[R70] Vahmani P, & Ban-Weiss GA (2016). Impact of remotely sensed albedo and vegetation fraction on simulation of urban climate in WRF-urban canopy model: A case study of the urban heat island in Los Angeles. Journal of Geophysical Research: Atmospheres, 121(4), 1511–1531. 10.1002/2015JD023718

[R71] Vahmani P, Luo X, Jones A, & Hong T (2022). Anthropogenic heating of the urban environment: An investigation of feedback dynamics between urban micro-climate and decomposed anthropogenic heating from buildings. Building and Environment, 213, 108841. 10.1016/j.buildenv.2022.108841

[R72] Wang J, Miao S, Doan Q-V, Chen F, Abolafia-Rosenzweig R, Yang L, (2023). Quantifying the impacts of high-resolution urban information on the urban thermal environment. Journal of Geophysical Research: Atmospheres, 128(6), e2022JD038048. 10.1029/2022JD038048

[R73] Wang Z, Bou-Zeid E, Au SK, & Smith JA (2011). Analyzing the sensitivity of WRF’s single-layer urban Canopy model to parameter uncertainty using advanced Monte Carlo simulation. Journal of Applied Meteorology and Climatology, 50(9), 1795–1814. 10.1175/2011JAMC2685.1

[R74] Xu Y, Vahmani P, Jones A, & Hong T (2024). Anthropogenic heat from buildings in Los Angeles County: A simulation framework and assessment. Sustainable Cities and Society, 107, 105468. 10.1016/j.scs.2024.105468

[R75] Yamamoto M, Kasai M, Okaze T, Hanaoka K, & Mochida A (2018). Analysis of climatic factors leading to future summer heatstroke risk changes in Tokyo and Sendai based on dynamical downscaling of pseudo global warming data using WRF. Journal of Wind Engineering and Industrial Aerodynamics, 183, 187–197. 10.1016/j.jweia.2018.10.001

[R76] Yang Z, Peng J, Jiang S, Yu X, & Hu T (2024). Optimizing building spatial morphology to alleviate human thermal stress. Sustainable Cities and Society, 106, 105386. 10.1016/j.scs.2024.105386

[R77] Yao Z, & Huang G (2023). Effects of land use changes across different urbanization periods on summer rainfall in the Pearl River Delta Core area. International Journal of Disaster Risk Science, 14(3), 458–474. 10.1007/s13753-023-00497-8

[R78] Yu E, Bai R, Chen X, & Shao L (2022). Impact of physical parameterizations on wind simulation with WRF V3.9.1.1 under stable conditions at planetary boundary layer gray-zone resolution: A case study over the coastal regions of North China. Geoscientific Model Development, 15(21), 8111–8134. 10.5194/gmd-15-8111-2022

[R79] Yu M, Chen X, Yang J, & Miao S (2021). A new perspective on evaluating high-resolution urban climate simulation with urban canopy parameters. Urban Climate, 38, 100919. 10.1016/j.uclim.2021.100919

[R80] Zhang J (2021). Modern Monte Carlo methods for efficient uncertainty quantification and propagation: A survey. WIREs Computational Statistics, 13(5), e1539. 10.1002/wics.1539

[R81] Zhang J, Li Y, Tao W, Liu J, Levinson R, Mohegh A, & Ban-Weiss G (2019). Investigating the urban air quality effects of cool walls and cool roofs in Southern California. Environmental Science & Technology, 53(13), 7532–7542. 10.1021/acs.est.9b00626 31125208

[R82] Zhang J, Mohegh A, Li Y, Levinson R, & Ban-Weiss G (2018). Systematic comparison of the influence of cool wall versus cool roof adoption on urban climate in the Los Angeles basin. Environmental Science & Technology, 52(19), 11188–11197. 10.1021/acs.est.8b00732 30157379

[R83] Zhao S, Chen Y, Zhang H, & Luo M (2024). Impacts of local climate zone mapping quality on urban near-surface air temperature simulation in WRF-UCM. Sustainable Cities and Society, 101, 105171. 10.1016/j.scs.2024.105171

[R84] Zhao Y, Zhong L, Ma Y, Fu Y, Chen M, Ma W, (2021). WRF/UCM simulations of the impacts of urban expansion and future climate change on atmospheric thermal environment in a Chinese megacity. Climatic Change, 169(3), 38. 10.1007/s10584-021-03287-7

[R85] Zhu D, & Ooka R (2023). WRF-based scenario experiment research on urban heat island: A review. Urban Climate, 49, 101512. 10.1016/j.uclim.2023.101512

