# Supplementary material for: Quantifying Urban Morphology-Induced Uncertainty in Urban Meteorology and Heat Stress Simulations in Southern California
Source: J Geophys Res Atmos. Author manuscript; Available in PMC 2026 Apr 18. (PMC13089901; doi:10.1029/2025jd045318)
Supplement: Supporting information S1 [file NIHMS2163775-supplement-Supporting_information_S1.docx]

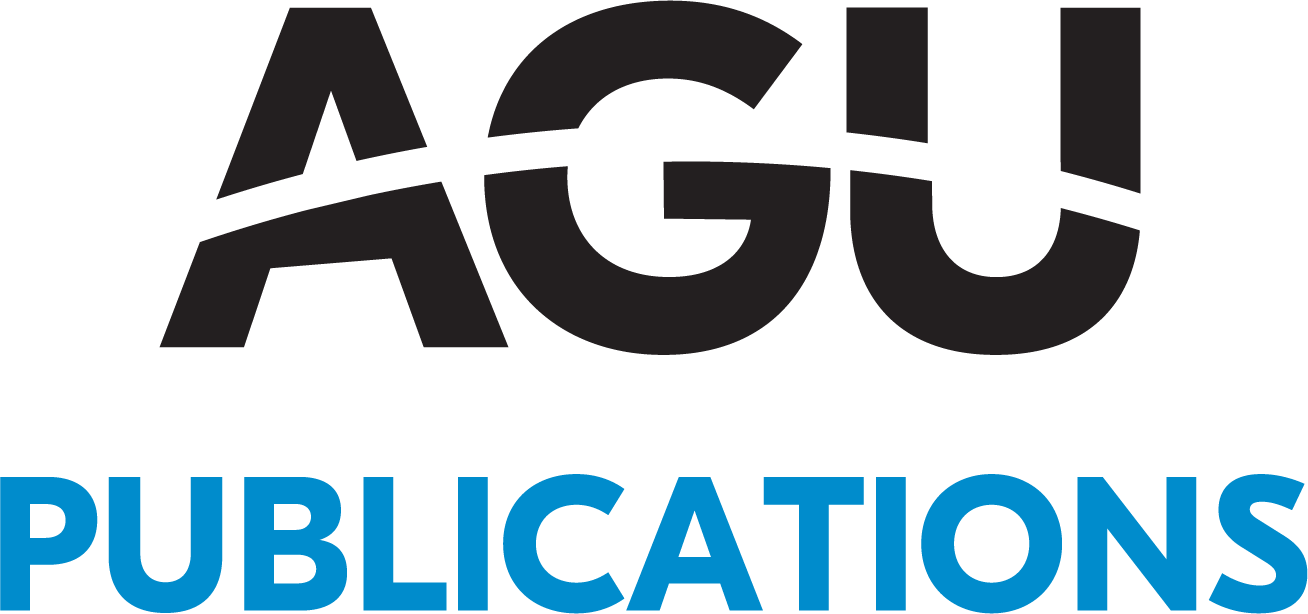


***Journal of Geophysical Research: Atmospheres***

Supporting information for

**Quantifying Urban Morphology-Induced Uncertainty in Urban Meteorology and Heat Stress Simulations in Southern California**

Hao Hu^1^, Xinyi Zhang^1^, Cenlin He^2^, Ellis Fertig^1^, Reza Zarrin^1^, Soroush E. Neyestani^1^, Jiachen Zhang^1,*^

^1^ Department of Civil and Environmental Engineering, University of Southern California, Los Angeles, CA, USA.

^2^ NSF National Center for Atmospheric Research (NCAR), Boulder, CO, USA

^*^**Correspondence to:**

Jiachen Zhang. [jiachen.zhang@usc.edu](mailto:jiachen.zhang@usc.edu)

**Contents of this file**

Text S1 to S5

Table S1 to S8

Figure S1 to S14

References

**Introduction**

This supporting information includes: the methodology for calculating urban morphological parameters in the domain 02 for WRF-SLUCM v. 4.6.1; the model evaluation metrics; all the tested physics schemes; detail of model configurations; LCZ land use data; PCE fitting performance; and additional PCE-based uncertainty quantification results not presented in the main text.

# Text S1. LCZ customized urban morphology parameters

Urban canopy morphological parameters (UMPs) (i.e., building height (H), roof width (R) and road width (W)) for each land use type is derived from real-world data from the Los Angeles Region Imagery Acquisition Consortium dataset (LARIAC 2014). LARIAC provides detailed county-wide building outline information, including building height, building shape, and location for each structure in Los Angeles County. Those parameters in the WRF Single layer urban canopy model (SLUCM) are calculated satisfying: (a) 2 × 𝐻/𝑅 as the ratio of gross wall area to roof area in LARIAC (2014) (Zhang et al., 2018) and (b) the ratio of road width to roof width (W/𝑅) matched to values of road area and roof area from LARIAC (2014).

Each 1 km × 1 km WRF grid cell is first classified into one LCZ land-use type based on the raw LCZ input data (Demuzere et al., 2022). We then assign an LCZ category for each building in the LARIAC dataset by identifying the LCZ class of the grid cell in which its centroid is located. For each LCZ land use type, building heights are averaged to compute a single representative building height H in the SLUCM model. Similarly, a representative value 𝑅 is assigned to each land use type as roof width, derived by aligning 2 × 𝐻/𝑅 with the ratio of the sum of wall area to the sum of roof area for all buildings of that type. The gross wall area for each building is calculated by multiplying its building height by its building perimeter, both from LARIAC (2014).

Road width W for each LCZ land use type is derived from the representative roof width R and the impervious surface fraction for that land use. Assuming that the impervious area only consists of roof area and road area, the total impervious area for each land use type is calculated as the product of the total land area and the impervious surface fraction for that class, and the total road area is calculated by subtracting the total roof area from the total impervious area. Finally, the representative road width W for each land use type is derived by the representative R for that class multiplied by the ratio of total road area to the total roof area for that class.

The default urban morphology parameters for LCZ urban land types and our customized parameters are presented in Table S4 and Table S5, respectively.

# Text S2. Model evaluation metrics

Assessment metrics commonly used in climate modeling are calculated based on AQS station observations within the South Coast Air Basin Region (SoCAB) and the WRF-SLUCM simulation results (Equation (1)-(4)).

All WRF–SLUCM outputs were first paired with hourly observations by matching each AQS station to the grid cell in which it is located. Evaluation metrics were then computed from the pooled set of all matched hourly pairs across all stations, i.e., using all stations and all hours simultaneously. This includes the mean bias (MB), mean absolute error (MAE), root mean square error (RMSE) and coefficient of determination ($R^{2}$). They are calculated as follows:

| $MB=\frac{1}{n}\sum_{i=1}^{n} \left( M_{i}-O_{i} \right)$ | (1) |
| --- | --- |
| $MAE=\frac{1}{n}\sum_{i=1}^{n} \left\vert\left( M_{i}-O_{i} \right) \right\vert$ | (2) |
| $RMSE=\sqrt{\frac{1}{n}\sum_{i=1}^{n} \left( M_{i}-O_{i} \right)^{2}}$ | (3) |
| $R^{2}=1-\frac{\sum_{i=1}^{n} \left( M_{i}-O_{i} \right)^{2}}{\sum_{i=1}^{n} \left( M_{i}-\bar{O} \right)^{2}}$ | (4) |

where n is the number of samples, $M_{i}$ is the *i*th simulation value, $O_{i}$ is the *i*th observation value, and $\bar{O}$ is the observation data's mean value. These metrics are used to evaluate the performance of WRF-SLUCM simulations and to compare the performance of different configurations.

# Text S3. Variation range of urban morphological parameters

In this study, we set the uncertainty range for the UMPs in WRF-SLUCM simulations in the Southern California region to ±50% based on two main considerations.

Firstly, when using LCZ land use data, defaulting UMPs are typically applied such as those provided for LCZ classes in the WRF model’s URBPARM.TBL. According to (Stewart & Oke, 2012), these LCZ land use types are estimated as range values for parameters. For example, the default building height for a given LCZ is typically the midpoint of that LCZ’s general height range, and the upper and lower bounds of the range are usually about ±50% of this midpoint as shown in Table S6. This means that the assumed “representative” building height could easily be 50% higher or lower while still falling within the typical range for that land-use class, indicating a substantial potential variability.

Secondly, even when we derive customized UMP values for a specific city using the real-world data, such as Los Angeles, we typically assign a single representative value to each urban land type or LCZ category for all grid cells, representing a structural uncertainty. This approach ignores the variation among different neighborhoods and individual buildings, inevitably introducing uncertainty into the model’s outputs. Our analysis using detailed building information for Los Angeles revealed considerable variability in urban morphology characteristics. For instance, the 10th and 90th percentile building heights in the city are roughly 50% and 150% of the mean building height, respectively (Table S7). Moreover, related parameters such as road width and roof width are derived from building height and thus exhibit similar variability.

These results indicate that while an average value can reasonably characterize the overall urban morphology, using a single mean value for an entire land-use category can lead to underestimation in some areas and overestimation in others. In summary, to account for both the uncertainty in default parameter values and the spatial variability of actual urban form, we adopt an uncertainty range of ±50% for all UMP values (assuming a uniform distribution within this range). This ±50% interval is meant to encapsulate the plausible variation in UMPs due to non-ideal parameter settings or the use of spatially uniform parameters, thereby allowing us to quantify the sensitivity of the WRF-SLUCM outputs to these uncertainties.

# Text S4. PCE methodology and training process

Polynomial Chaos Expansion (PCE) is an efficient uncertainty quantification technique. Generally, the PCE approach formulates the relationship between the system output $\mathbf{f}\left( \mathbf{X} \right)$ (in our study, $\mathbf{f}\left( \mathbf{X} \right)$ can be 2-m air temperature ($T_{2}$), urban canopy temperature ($T_{C}$), relative humidity (RH), wind speed, planetary boundary layer height (PBLH), urban area net shortwave radiation ($SW_{\mathrm{NET}}$), urban area net longwave radiation ($LW_{\mathrm{NET}}$), urban area net radiation ($R_{\mathrm{NET}}$)) and each random input $\mathbf{X}$ (in our study, it is building height, road width and roof width) using a series of orthogonal polynomial basis functions $\boldsymbol{\Psi}_{\boldsymbol{\alpha}}$, which can be written as equation (1):

| $\mathbf{f}\left( \mathbf{X} \right)\approx\sum_{\alpha=0}^{M} \mathbf{c}_{\boldsymbol{\alpha}}\boldsymbol{\Psi}_{\boldsymbol{\alpha}}(\mathbf{X})$ | (1) |
| --- | --- |

where $\boldsymbol{\alpha}$ denotes a multi-dimensional set of polynomial indices and $\mathbf{c}_{\boldsymbol{\alpha}}$​ are the corresponding expansion coefficients to be determined. In this study, Legendre polynomials are employed due to their suitability for uniformly distributed random variables.

The selection of the polynomial degree **M** in the PCE method is a critical consideration, as it directly influences the balance between model accuracy and computational feasibility. Given that we only generated 100 samples, the tested polynomial degrees ranged from 1 to 3 in this study.

After fixing the total polynomial degree **M**, we run the model at 100 distinct input samples $\mathbf{X}$, and collect the corresponding outputs $\mathbf{Y}$. We then build the design matrix $\boldsymbol{\Psi}_{\boldsymbol{\alpha}}(\mathbf{X})$. The coefficient vector $\mathbf{c}_{\boldsymbol{\alpha}}$is obtained by minimizing the sum of squared residuals as shown in equation (2):

| $\mathbf{c}_{\boldsymbol{\alpha}}=\arg\min_{\alpha} \left\vert{\vert\mathbf{c}_{\boldsymbol{\alpha}}\boldsymbol{\Psi}}_{\boldsymbol{\alpha}}\left( \mathbf{X} \right)-Y \right\vert\vert_{2}^{2}$ | (2) |
| --- | --- |

In the implementation, this regression is performed in one step via a scikit-learn Pipeline (imputation → scaling → polynomial feature expansion → Linear Regression), which directly returns the intercept and coefficients $\mathbf{c}_{\boldsymbol{\alpha}}$ for use in subsequent PCE analysis.

Once the coefficients are determined, we calculate the statistical characteristics, including mean value and standard deviations (SD) of the uncertainty system output by the truncated PCE model as shown in equation (3) – (4),

| $E\left[ f\left( X \right) \right]=c_{0}$ | (3) |
| --- | --- |
| $\mathrm{SD}\left[ f\left( X \right) \right]=sqrt(\sum_{\alpha=0}^{M} \mathbf{c}_{\boldsymbol{\alpha}}^{2}\left\vert\left\vert\boldsymbol{\Psi}_{\boldsymbol{\alpha}}\left( \mathbf{X} \right) \right\vert\right\vert^{2})$ | (4) |

where $E\left[ f\left( X \right) \right]$ represents the mean value of target variables and $\mathrm{SD}\left[ f\left( X \right) \right]$ represents the standard deviation of target variables. While $\left| \left| \boldsymbol{\Psi}_{\boldsymbol{\alpha}}\left( \mathbf{X} \right) \right| \right|^{2}$ represents the squared norm of $\boldsymbol{\Psi}_{\boldsymbol{\alpha}}\left( \mathbf{X} \right)$, which is calculated as equation (5).

| $\left\vert\left\vert\boldsymbol{\Psi}_{\boldsymbol{\alpha}}\left( \mathbf{X} \right) \right\vert\right\vert^{2}=\int_{\Omega} \boldsymbol{\Psi}_{\boldsymbol{\alpha}}\left( \mathbf{X} \right)^{2}p(X)dX$ | (5) |
| --- | --- |

In equation, $p(X)$ is the probability density function (PDF) of the random variable X. For Legendre, $p\left( X \right)$ = 1/2. Thus,

| $\left\vert\left\vert\boldsymbol{\Psi}_{\boldsymbol{\alpha}}\left( \mathbf{X} \right) \right\vert\right\vert^{2}=\frac{2}{2\alpha+1}\times\frac{1}{2}$ | (6) |
| --- | --- |

To evaluate the predictive performance of the PCE surrogate model, we compute the coefficient of determination, which quantifies the proportion of variance in the output variable that is explained by the surrogate model.

# Text S5. Sobol Index calculation

In this study, input variables are first normalized according to their respective ranges, and then orthogonal Legendre polynomial bases are constructed. The expansion coefficients are determined using linear regression. With the resulting PCE model, we compute both uncertainty measures and Sobol sensitivity indices. Sobol indices quantify the contributions of individual inputs or their interactions to the total output variance and are formally defined as:

| $S_{i}=\frac{\sum_{\alpha\in A_{i}} \mathbf{c}_{\boldsymbol{\alpha}}^{2}\left\vert\left\vert\boldsymbol{\Psi}_{\boldsymbol{\alpha}}\left( \mathbf{X} \right) \right\vert\right\vert^{2}}{Var[f(X)]}$ | (7) |
| --- | --- |
| $S_{\mathrm{ij}}=\frac{\sum_{\alpha\in A_{\mathrm{ij}}} \mathbf{c}_{\boldsymbol{\alpha}}^{2}\left\vert\left\vert\boldsymbol{\Psi}_{\boldsymbol{\alpha}}\left( \mathbf{X} \right) \right\vert\right\vert^{2}}{Var[f(X)]}$ | (8) |

where $\mathbf{S}_{\mathbf{i}}$ represents the first-order Sobol index (the direct effect of a single parameter $X_{i}$ on the output), while $\mathbf{S}_{\mathbf{ij}}$ denotes the second-order Sobol index (capturing interactions between two parameters). $A_{i}$ is the set of multi-indices where only the $i\mathrm{th}$ element is nonzero, and $A_{\mathrm{ij}}$​ is the set where only the $i\mathrm{th}$ and $j\mathrm{th}$ elements are nonzero.

By employing the PCE approach described here, this research systematically identifies critical input parameters (e.g., urban morphological parameters) and their contributions to the uncertainties and sensitivities of WRF-SLUCM urban climate simulation outputs. These findings can guide the optimization of model parameters and direct future efforts in data collection.

# Table S1. Summary of WRF–SLUCM Studies in Southern California and their UCM Data Sources and Simulation Length.

| **Paper** | **Research Topic** | **Model Used** | **UCM data source** | **Simulation Days** |
| --- | --- | --- | --- | --- |
| (P. Vahmani & Hogue, 2015) | Urban irrigation | WRF-SLUCM | NUDAPT (where available) and default | 180 |
| (P. Vahmani & Ban-Weiss, 2016b) | Albedo and vegetation fraction | WRF-SLUCM | NUDAPT (where available) and default | 5 |
| (P. Vahmani & Ban-Weiss, 2016a) | Drought-tolerant vegetation | WRF-SLUCM | NUDAPT (where available) and default | 33 |
| (Pouya Vahmani & Jones, 2017) | Cool roofs | WRF-SLUCM | NUDAPT (where available) and default | 165 |
| (P Vahmani et al., 2019) | Climate change, population and urban heat mitigation | WRF-SLUCM | NUDAPT (where available) and default | 165 |
| (Luo et al., 2020) | Anthropogenic heat | WRF-SLUCM | NUDAPT (where available) | 15 |
| (P. Vahmani et al., 2022) | Climate change and irrigation | WRF-SLUCM | NUDAPT (where available) | ~150 |
| (Pouya Vahmani et al., 2022) | Anthropogenic heating | WRF-SLUCM | NUDAPT (where available) | 45 |
| (Xu et al., 2024) | Anthropogenic Heat | WRF-SLUCM | NUDAPT (where available) | 31 |
| (Zhang et al., 2018) | Cool roof and cool wall | WRF-SLUCM | NUDAPT (where available) and customized UMPs | 12-14 |
| (Schlaerth et al., 2023) | Albedo and urban greening | WRF-SLUCM | NUDAPT (where available) | 31 |
| (P Vahmani et al., 2016) | Urbanization and cool roofs | WRF-SLUCM | NUDAPT (where available) | 33 |
| (Li et al., 2019) | Urbanization | WRF-SLUCM | NUDAPT (where available) and customized UMPs | 10 |
| (Zhang et al., 2019) | Cool roof and cool wall | WRF-SLUCM | Customized and NUDAPT (where available) and customized UMPs | 14 |
| (Kalkstein et al., 2022) | Tree cover and albedo | WRF-SLUCM | Default | 11-12 |

# Table S2. Summary of the physics schemes and options number used in the WRF-SLUCM simulations for selecting the best optimal combination of physics schemes and quantifying the associated uncertainty represented by the standard deviation.

| **Physics scheme** | **Option (Option number)** |
| --- | --- |
| Microphysics | Morrison 2-moment scheme (10) |
|  | WSM 6-class graupel scheme (6) |
|  | Thompson graupel scheme (8) |
| Cumulus Parameterization | Modified Kain-Fritsch scheme with trigger function based on PDFs (10) |
| Shortwave/Longwave Radiation | RRTMG (4/4) |
| Planetary Boundary Layer (PBL)/ Surface Layer Physics (SLP) | QNSE-EDMF/ QNSE (4/4) |
|  | UW-TKE (9)/ Revised MM5 Scheme (1) |
|  | ACM2 (7) / Revised MM5 Scheme (1) |
|  | YSU (1)/ Revised MM5 Scheme (1) |
| Land Surface Mode | unified Noah land-surface model (2) |
|  | Noah-MP Land Surface Model (4) |

# Table S3. Tested combinations of physics schemes sets. Simulations M1-M24 differ in their microphysics, PBL, SLP, and land surface model options (see Table S2 for details). The asterisk marks the final selected schemes for baseline simulation in the main text.

| No | Microphysics | PBL | SLP | Land Surface |
| --- | --- | --- | --- | --- |
| M1* | 10 | 4 | 4 | 2 |
| M2 | 6 | 4 | 4 | 2 |
| M3 | 8 | 4 | 4 | 2 |
| M4 | 10 | 9 | 1 | 2 |
| M5 | 6 | 9 | 1 | 2 |
| M6 | 8 | 9 | 1 | 2 |
| M7 | 10 | 7 | 1 | 2 |
| M8 | 6 | 7 | 1 | 2 |
| M9 | 8 | 7 | 1 | 2 |
| M10 | 10 | 1 | 1 | 2 |
| M11 | 6 | 1 | 1 | 2 |
| M12 | 8 | 1 | 1 | 2 |
| M13 | 10 | 4 | 4 | 4 |
| M14 | 6 | 4 | 4 | 4 |
| M15 | 8 | 4 | 4 | 4 |
| M16 | 10 | 9 | 1 | 4 |
| M17 | 6 | 9 | 1 | 4 |
| M18 | 8 | 9 | 1 | 4 |
| M19 | 10 | 7 | 1 | 4 |
| M20 | 6 | 7 | 1 | 4 |
| M21 | 8 | 7 | 1 | 4 |
| M22 | 10 | 1 | 1 | 4 |
| M23 | 6 | 1 | 1 | 4 |
| M24 | 8 | 1 | 1 | 4 |

# Table S4. Default urban morphological parameters for LCZ land use types in Southern California.

|  | LCZ1 | LCZ2 | LCZ3 | LCZ5 | LCZ6 | LCZ8 |
| --- | --- | --- | --- | --- | --- | --- |
| Building Height (m) | 37.5 | 17.5 | 6.5 | 17.5 | 6.5 | 6.5 |
| Building Height std. | 4.0 | 3.0 | 1.0 | 1.0 | 1.0 | 1.0 |
| Roof Width (m) | 22.2 | 22.0 | 9.6 | 26.25 | 13.0 | 28.9 |
| Road Width (m) | 20.0 | 14.0 | 5.2 | 35.0 | 13.0 | 32.5 |
| LCZ 1~11 represents: Comp High-Rise, Comp Mid-Rise, Comp Low-Rise, Op H-Rise, Op M-Rise, Op L-Rise, Lightweight L-Rise, Large L-Rise, Sparsely Built, Heavy Indus Asphalt. The missing LCZ land types in the table indicate that these land types are not present in Southern California. The distribution of land use types in our study region in Southern California is shown in Figure S14. | | | | | | |

# Table S5. Customized urban morphological parameters for LCZ land use types based on real-world in Southern California.

|  | LCZ1 | LCZ2 | LCZ3 | LCZ5 | LCZ6 | LCZ8 |
| --- | --- | --- | --- | --- | --- | --- |
| Building Height (m) | 58.79 | 11.89 | 5.17 | 6.10 | 5.34 | 5.82 |
| Building Height std. | 60.58 | 15.34 | 2.61 | 2.42 | 2.49 | 4.29 |
| Roof Width (m) | 16.52 | 8.14 | 5.27 | 5.91 | 5.78 | 7.37 |
| Road Width (m) | 15.71 | 8.48 | 9.32 | 30.60 | 7.07 | 19.24 |
| LCZ 1~11 represents: Comp High-Rise, Comp Mid-Rise, Comp Low-Rise, Op H-Rise, Op M-Rise, Op L-Rise, Lightweight L-Rise, Large L-Rise, Sparsely Built, Heavy Indus Asphalt. The missing LCZ land types in the table indicate that these land types are not present in Southern California. The distribution of land use types in our study region in Southern California is shown in Figure S14. | | | | | | |

# Table S6. Default building height ranges (m) and mean values by LCZ types provided by (Stewart & Oke, 2012).

| LU type | Minimum | Maximum | Mean |
| --- | --- | --- | --- |
| LCZ1 | 25 | 50 | 37.5 |
| LCZ2 | 10 | 25 | 17.5 |
| LCZ3 | 3 | 10 | 6.5 |
| LCZ5 | 10 | 25 | 17.5 |
| LCZ6 | 3 | 10 | 6.5 |
| LCZ8 | 3 | 10 | 6.5 |

# Table S7. Statistical summary of building heights (10th, 90th percentiles and mean) in meter by land use type in Los Angeles

| LU type | Count | 10th percentile | 90th percentile | Mean |
| --- | --- | --- | --- | --- |
| LCZ1 | 1 | 7.00 | 148.15 | 58.79 |
| LCZ2 | 8 | 3.74 | 27.04 | 11.89 |
| LCZ3 | 448 | 2.89 | 8.02 | 5.17 |
| LCZ5 | 1 | 3.25 | 9.50 | 6.10 |
| LCZ6 | 4232 | 3.10 | 8.21 | 5.34 |
| LCZ8 | 1323 | 3.08 | 9.14 | 5.82 |

# Table S8. LU_INDEX code list and meanings. Values 1–17 are standard WRF MODIS/USGS categories; 51–59 are LCZ urban classes. Used for urban grid-cell classification in this work.

| LU_INDEX | Mean |
| --- | --- |
| 1 | Evergreen Needleleaf Forest |
| 5 | Mixed Forests |
| 6 | Closed Shrublands |
| 7 | Open Shrublands |
| 8 | Woody Savanna |
| 9 | Savanna |
| 10 | Grasslands |
| 11 | Permanent Wetlands |
| 12 | Croplands |
| 13 | Urban/Built-up (MODIS) |
| 16 | Barren/Sparse |
| 17 | Water |
| 51 | LCZ 1-Compact High-Rise |
| 52 | LCZ 2- Compact Mid-Rise |
| 53 | LCZ 3-Compact Low-Rise |
| 55 | LCZ 5-Open Mid-Rise |
| 56 | LCZ 6-Open Low-Rise |
| 58 | LCZ 8-Large Low-Rise |
| 59 | LCZ 9-Sparsely Built |


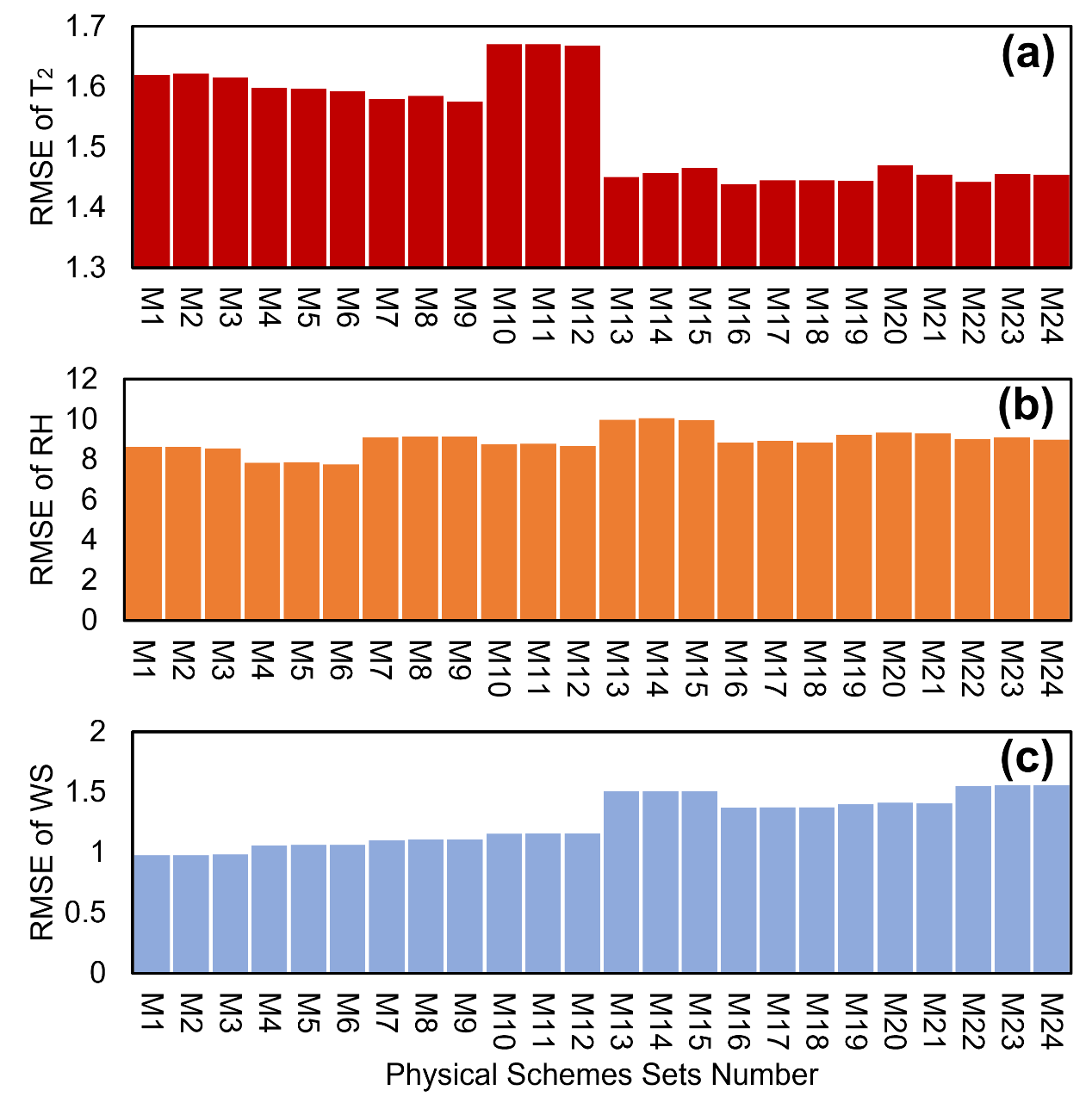


# Figure S1. Model evaluation in root mean square error (RMSE) of (a) temperature at 2 meters (T₂), (b) relative humidity (RH), and (c) wind speed (WS) for 24 WRF–SLUCM physics scheme configurations (M1–M24). Definitions of M1–M24 are provided in Table S3. Scheme M1 was selected as the baseline configuration in the main body. Detailed evaluation methods are provided in the Supporting Information Text S2.


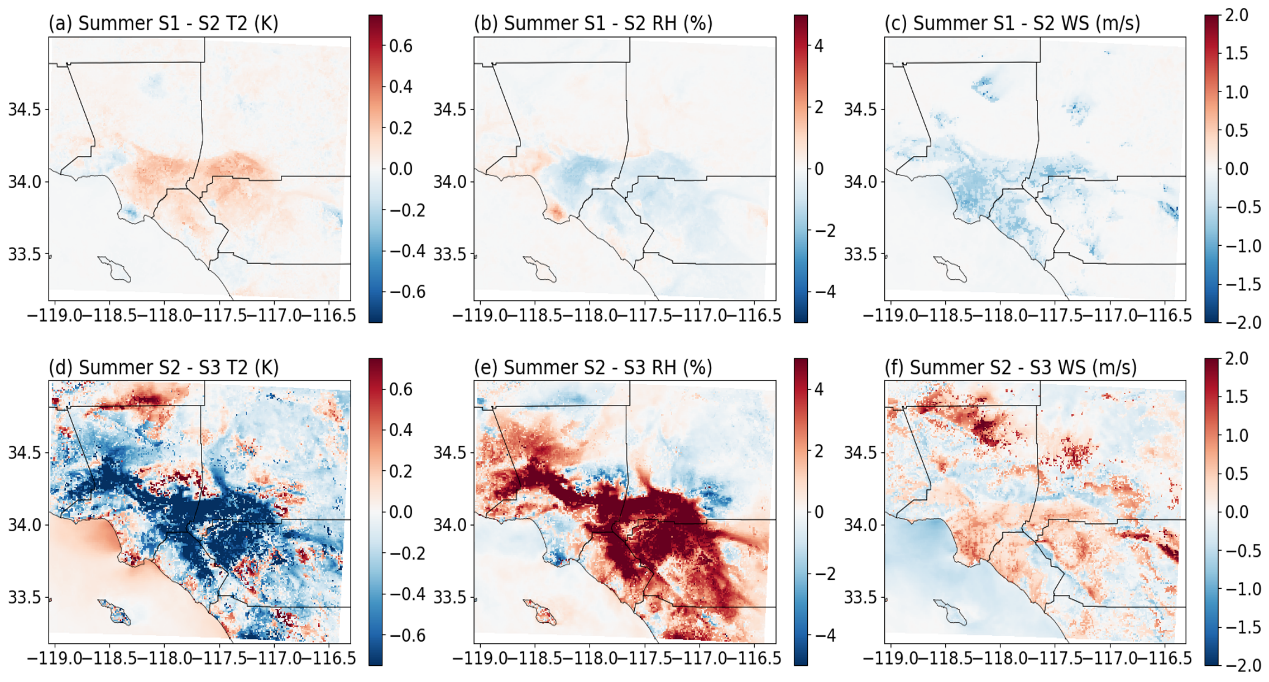


# Figure S2. Summer-mean spatial differences from WRF–SLUCM across two configurations: LCZ_LA (S1) and LCZ_Default (S2). Panels map differences in (a) 2-m air temperature (T₂), (b) relative humidity (RH), and (c) wind speed (WS).


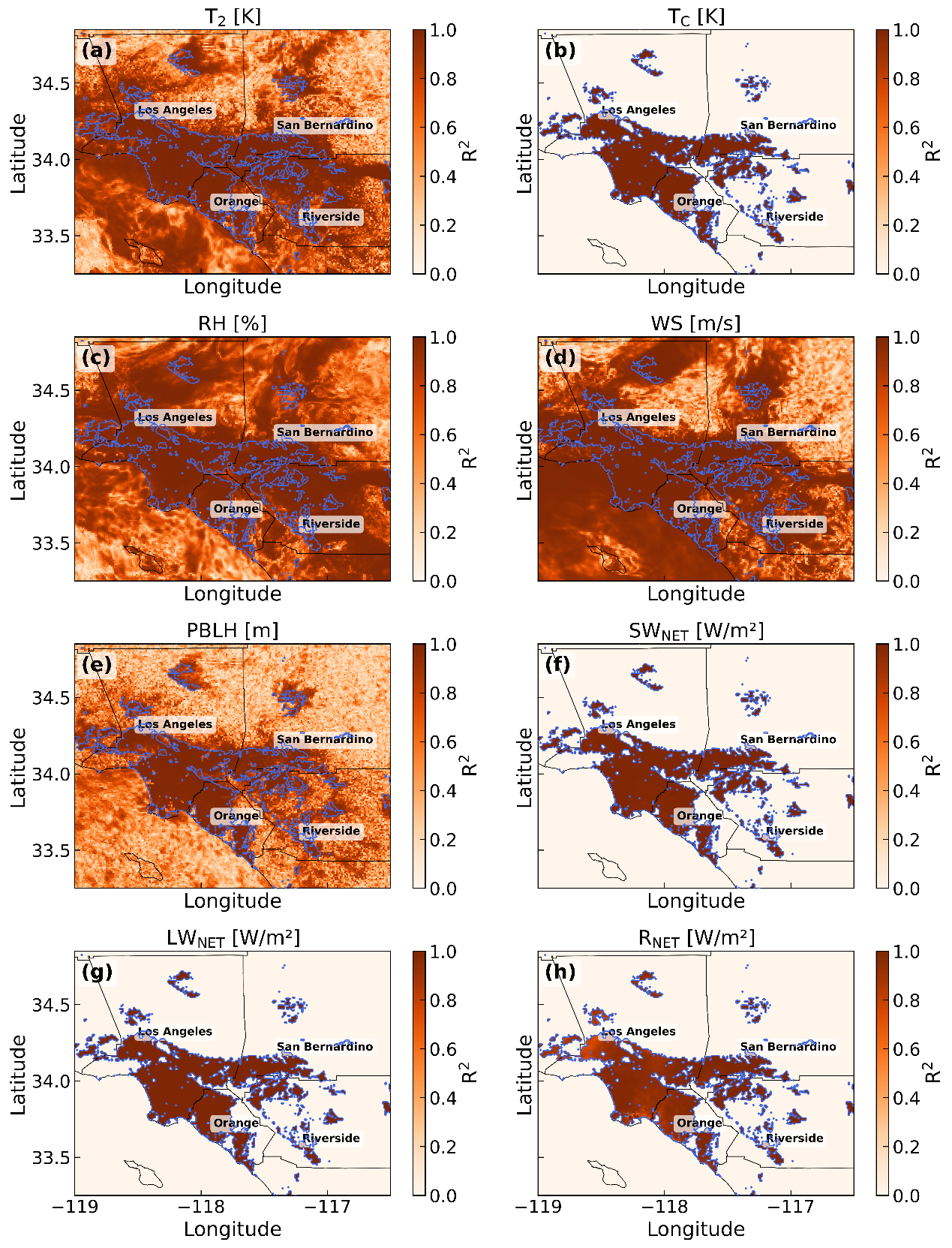


# Figure S3. Coefficient of determination (R²) for the PCE fit for (a) 2-m air temperature (T₂), (b) urban canopy temperature (T_C_), (c) relative humidity (RH), (d) 10-m wind speed (WS), (e) planetary boundary layer height (PBLH), (f) net shortwave radiation (SW_NET_), (g) net longwave radiation (LW_NET_), and (h) net all-wave radiation (R_NET_). Black outlines and labels denote counties in the South Coast Air Basin (Los Angeles, Orange, Riverside, and San Bernardino). Blue lines represent the urban boundaries derived from LCZ land use data.


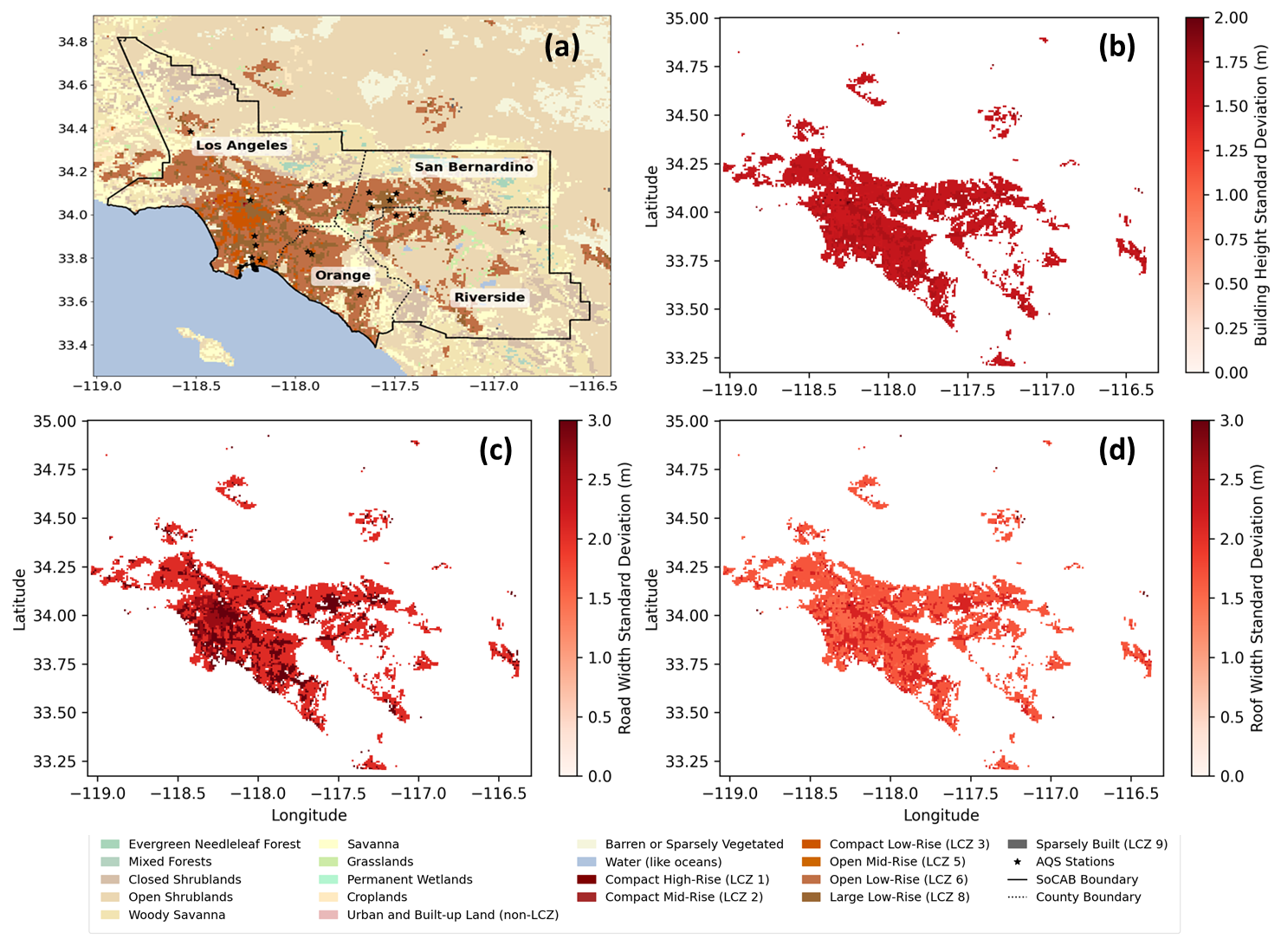


# Figure S4. (a) Local Climate Zone (LCZ)–based land-use map for the South Coast Air Basin, where indices 51–59 correspond to urban LCZ classes. Spatial distribution of standard deviations of (b) building height, (c) road width, and (d) roof width, which are used as perturbations for uncertainty quantification of simulations over Southern California.


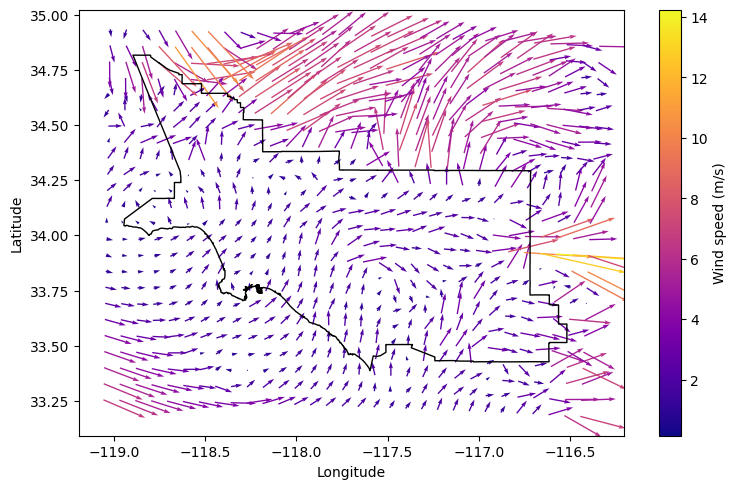


# Figure S5. Daily mean 10m wind vectors over the South Coast Air Basin, with arrow colors indicating wind speed (m/s) and black lines showing the SoCAB boundaries.


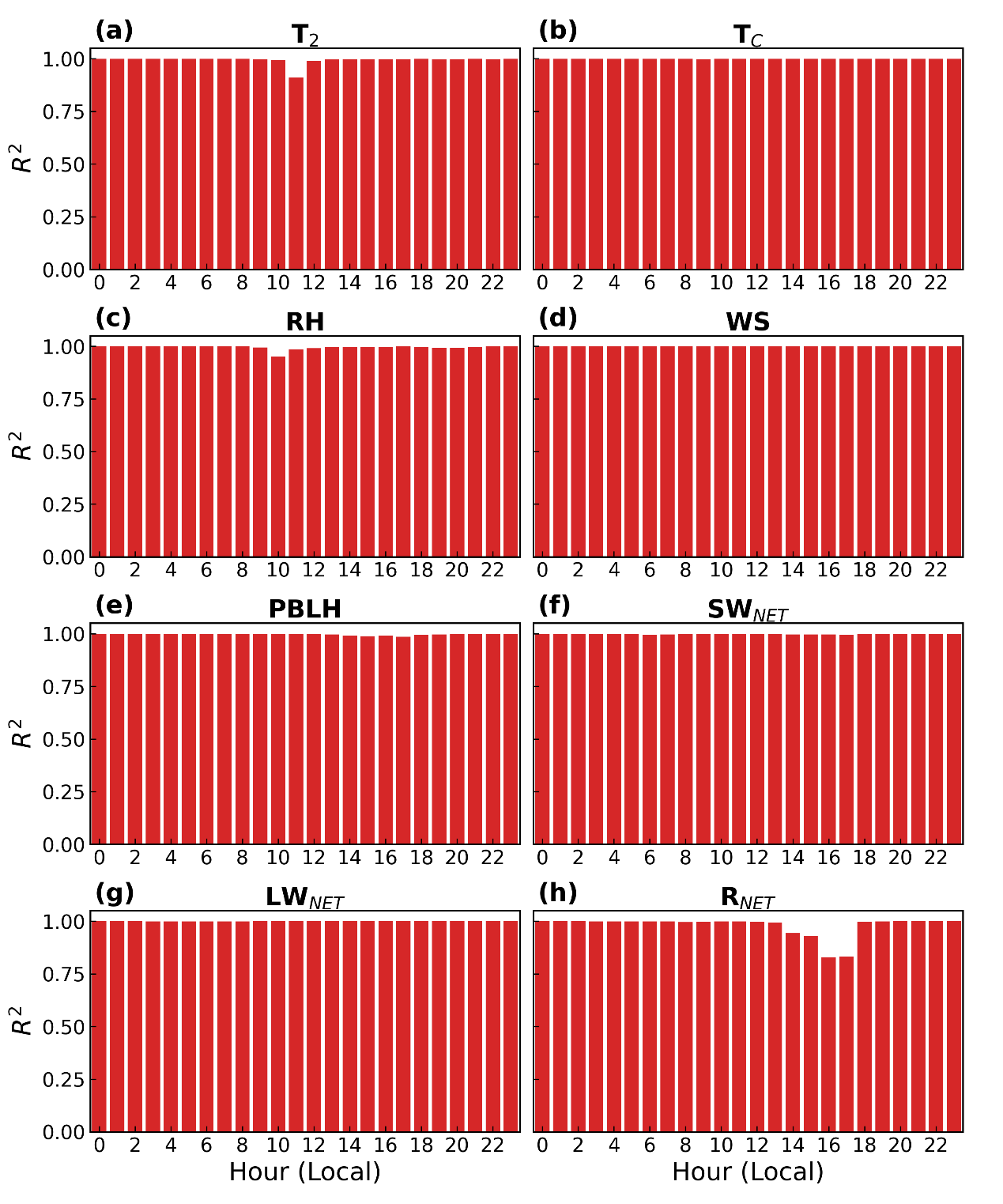


# Figure S6. Hourly coefficient of determination (R²) values for PCE surrogate models of eight WRF-SLUCM output variables during a summer simulation. Each bar represents the 5-cross fold R² between the PCE-predicted and original WRF outputs at each local hour (0–23): (a) 2-meter air temperature (T₂), (b) urban canopy temperature (TC), (c) relative humidity (RH), (d) wind speed (WS), (e) planetary boundary layer height (PBLH), (f) urban net shortwave radiation (SWNET), (g) urban net longwave radiation (LWNET), and (h) urban total net radiation (RNET). The consistently high R² values (mostly above 0.95) across all hours demonstrate the strong predictive performance of the PCE surrogate models for representing hourly variations in each variable


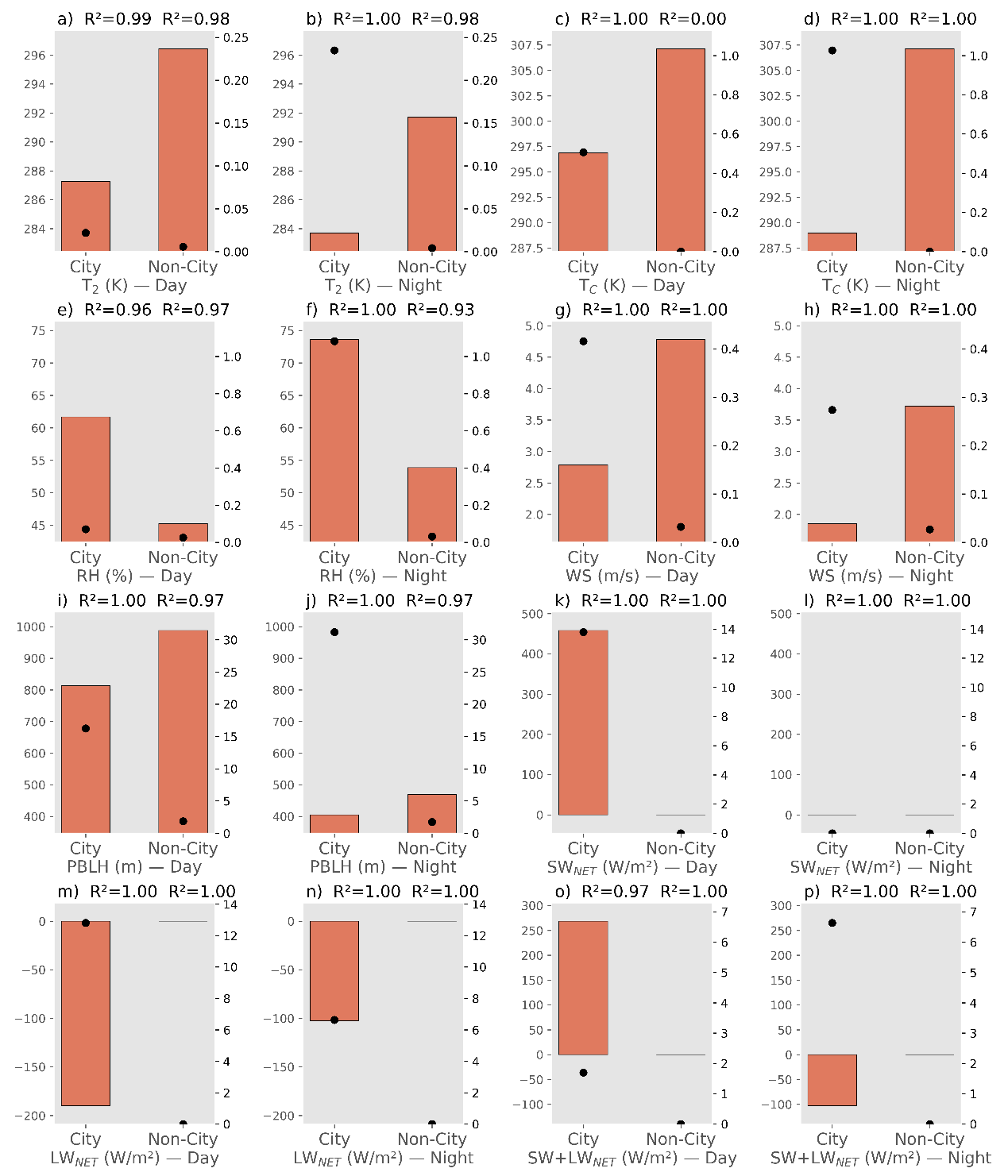


# Figure S7. PCE-based uncertainty analysis in WRF–SLUCM outputs for urban (city) and non-urban (non-city) grid cells average during summer daytime and nighttime. Each subplot presents the mean value (bar, left y-axis) and standard deviation (black dot, right y-axis) from trained PCE surrogate model with varying urban morphology parameter combinations. Panel headers report the coefficient of determination (R²) of the surrogate fit. Variables include: (a–b) T₂, (c–d) T_C_, (e–f) RH, (g–h) wind speed, (i–j) PBLH, (k–l) SW_NET_, (m–n) LW_NET_, and (o–p) R_NET_.


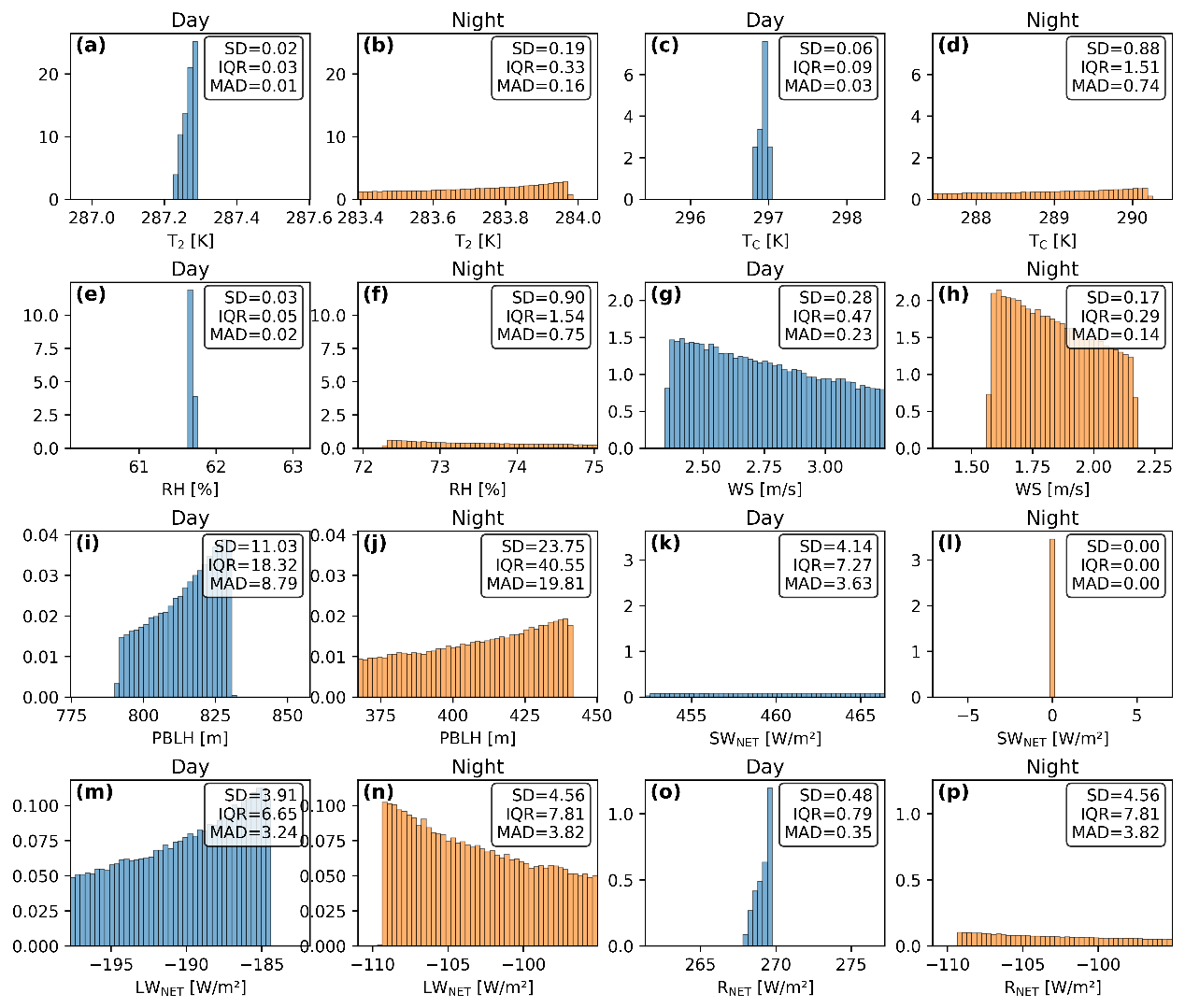


# Figure S8. Distribution of urban-mean outputs in domain 02 from 1,000,000 Monte Carlo simulations only varying building height. These variables include (a–b) T₂, (c–d) T_C_, (e–f) RH, (g–h) wind speed, (i–j) PBLH, (k–l) SW_NET_, (m–n) LW_NET_, (o–p) R_NET_. Blue bars represent daytime averages, and orange bars represent nighttime averages. Blue bars indicate daytime means and orange bars indicate nighttime means. Insets report the standard deviation (SD), interquartile range (IQR), and median absolute deviation (MAD) of outputs.


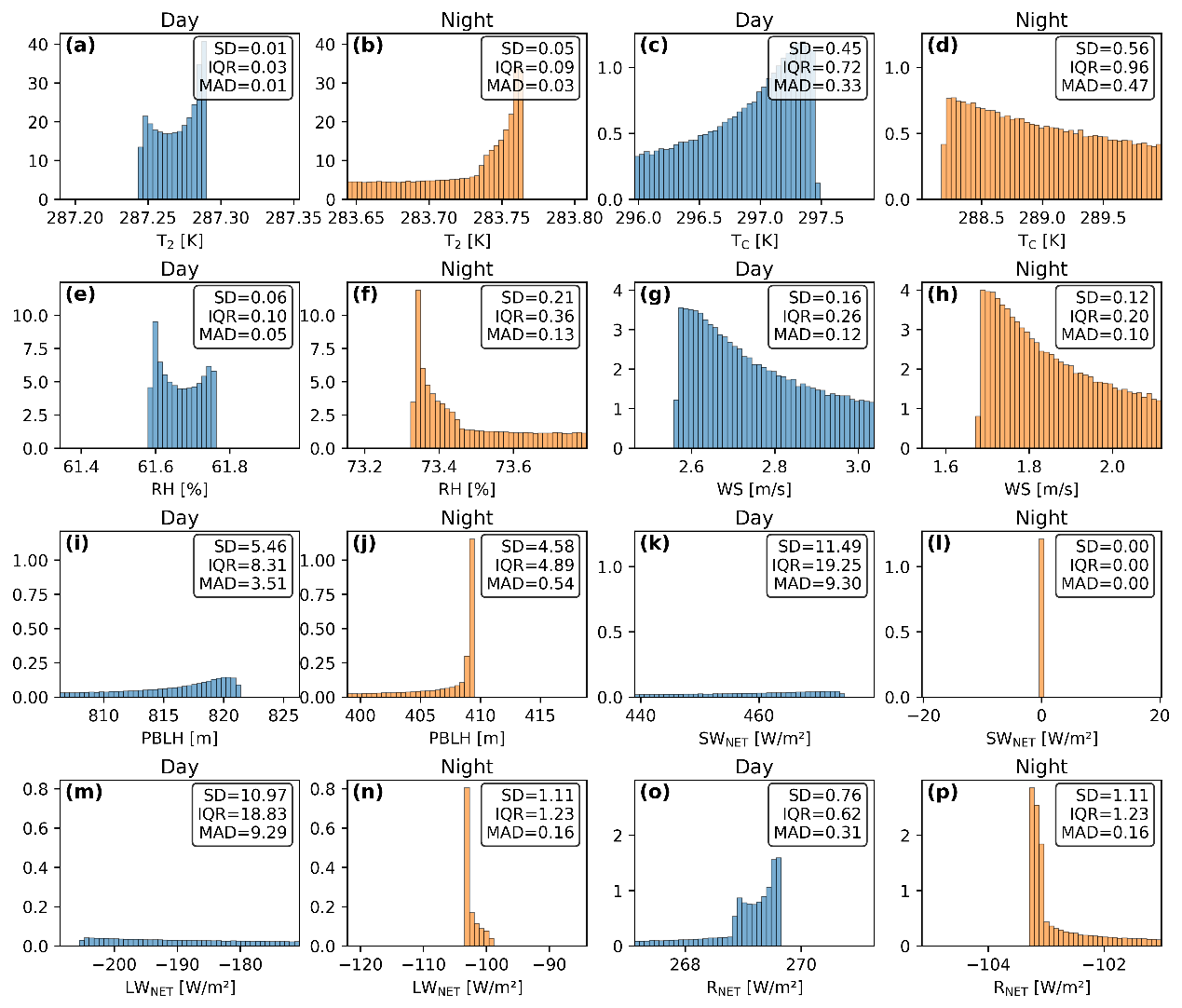


# Figure S9. Distribution of urban-mean outputs in domain 02 from 1,000,000 Monte Carlo simulations only varying roof width. These variables include (a–b) T₂, (c–d) T_C_, (e–f) RH, (g–h) wind speed, (i–j) PBLH, (k–l) SW_NET_, (m–n) LW_NET_, (o–p) R_NET_. Blue bars represent daytime averages, and orange bars represent nighttime averages. Insets report the standard deviation (SD), interquartile range (IQR), and median absolute deviation (MAD) of outputs.


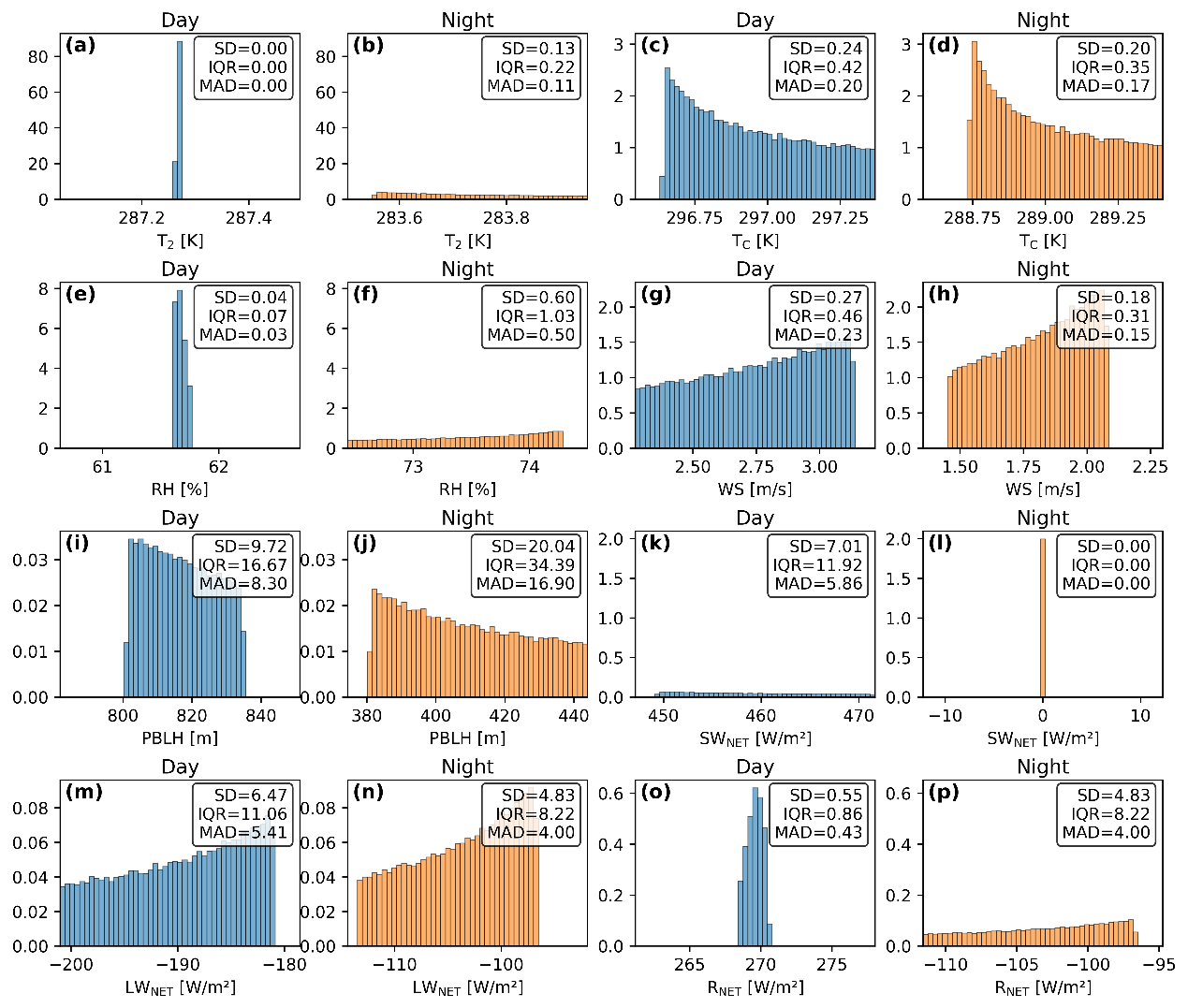


# Figure S10. Distribution of urban-mean outputs in domain 02 from 1,000,000 Monte Carlo simulations only varying road width. These variables include (a–b) T₂, (c–d) T_C_, (e–f) RH, (g–h) wind speed, (i–j) PBLH, (k–l) SW_NET_, (m–n) LW_NET_, (o–p) R_NET_. Blue bars represent daytime averages, and orange bars represent nighttime averages. Insets report the standard deviation (SD), interquartile range (IQR), and median absolute deviation (MAD) of outputs.


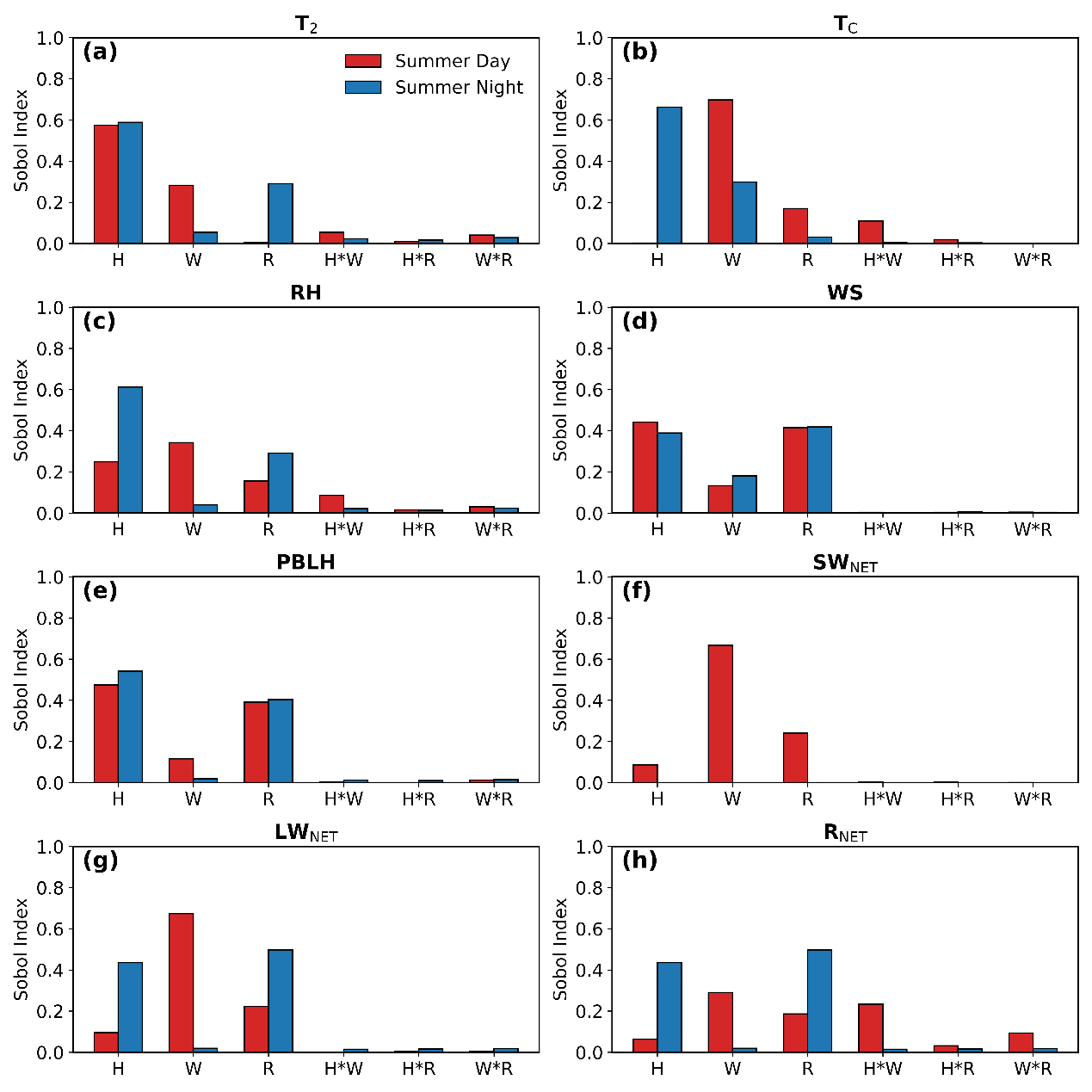


# Figure S11. Sobol Index from urban morphology parameters, including building height (H), road width (W), and roof width (R) to all WRF-SLUCM output variables ($\mathbf{T}_{\mathbf{2}}$, $\mathbf{T}_{\mathbf{C}}$, RH, WS, PBLH, $\mathbf{S}\mathbf{W}_{\mathbf{NET}}$, $\mathbf{L}\mathbf{W}_{\mathbf{NET}}$, and $\mathbf{R}_{\mathbf{NET}}$), averaged over urban areas. Individual variables represent their individual effects, while the multiplication of two variables indicates their interaction effects.


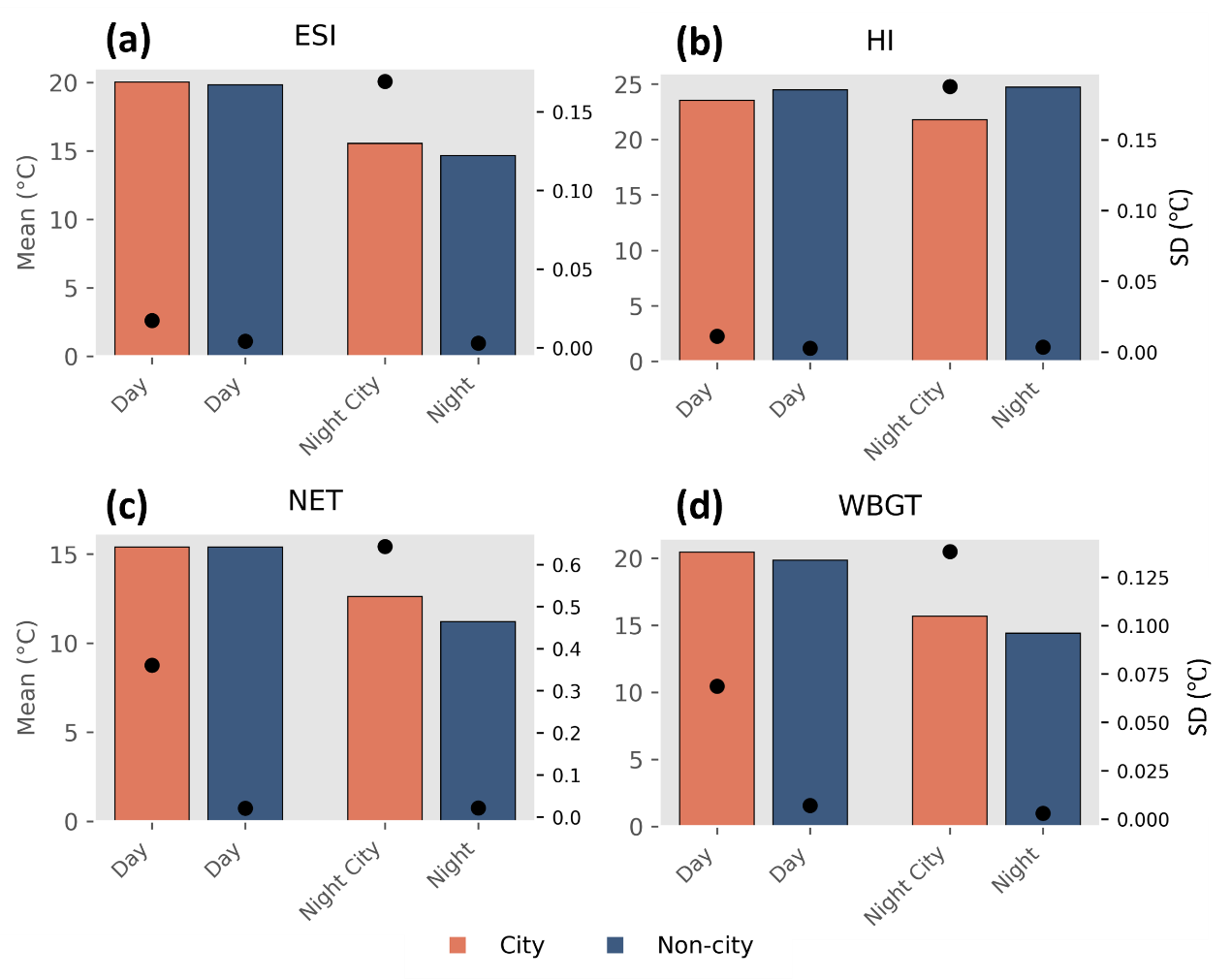


# Figure S12. Mean values (bars) and standard deviations (black dots) of four heat stress indicators: (a) ESI, (b) HI, (c) NET, and (d) WBGT, based on the results of Polynomial Chaos Expansion (PCE). The metrics are shown separately for daytime and nighttime periods, and for urban (city) and non-urban (non-city) grid cells. The standard deviation represents the uncertainty associated with UMP variability in the WRF-SLUCM simulations.


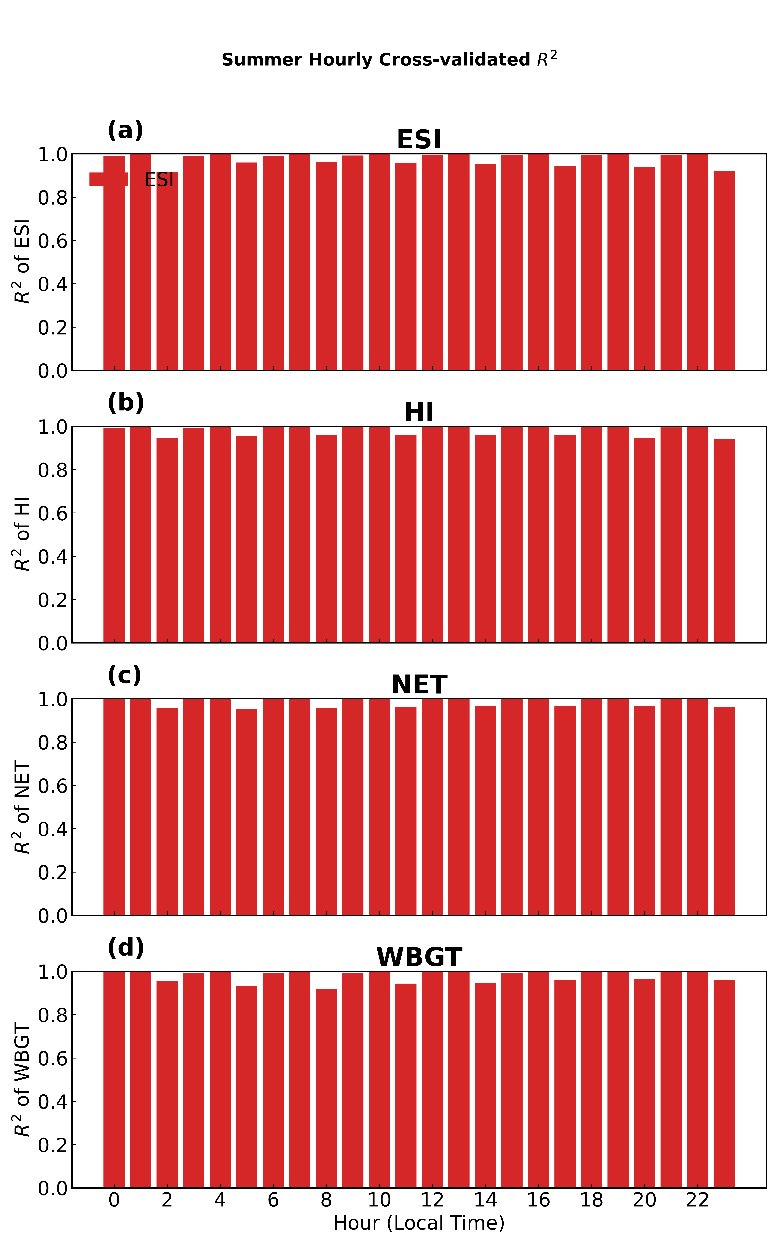


# Figure S13. Hourly coefficients of determination (R²) for PCE surrogate models of four heat-stress indicators during the summer period. Bars show the 5-fold cross-validated R² between PCE predictions and the corresponding indicator at each local hour (0–23): (a) ESI, (b) HI, (c) NET, and (d) WBGT. The consistently high R² values (generally >0.95) indicate strong surrogate fidelity in capturing hourly variability.


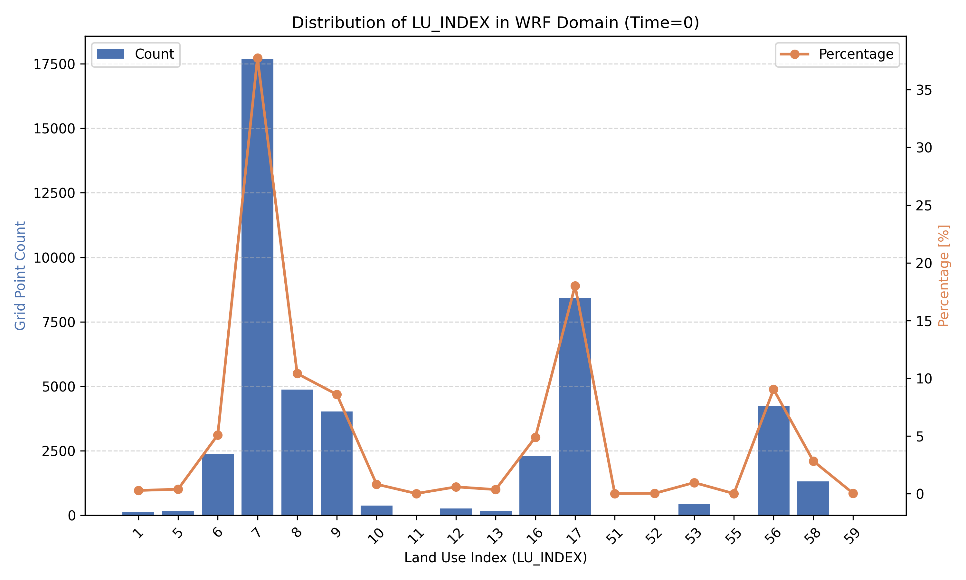


# Figure S14. Distribution of land-use categories (LU_INDEX) within the Southern California study domain. Bars show the number of grid cells in each category (left axis), and the overlaid line shows the percentage of total grid cells (right axis). This distribution is based on the WRF-SLUCM domain setup and LCZ land use classification applied in our study. The specific land use type represented by each LU_INDEX is provided in Table S8.

# References

Demuzere, M., Argüeso, D., Zonato, A., & Kittner, J. (2022). W2W: A Python package that injects WUDAPT’s LocalClimate Zone information in WRF. *Journal of Open Source Software*, *7*(76), 4432. https://doi.org/10.21105/joss.04432

Kalkstein, L. S., Eisenman, D. P., de Guzman, E. B., & Sailor, D. J. (2022). Increasing trees and high-albedo surfaces decreases heat impacts and mortality in Los Angeles, CA. *International Journal of Biometeorology*, *66*(5), 911–925. https://doi.org/10.1007/s00484-022-02248-8

Li, Y., Zhang, J., Sailor, D. J., & Ban-Weiss, G. A. (2019). Effects of urbanization on regional meteorology and air quality in Southern California. *Atmospheric Chemistry and Physics*, *19*(7), 4439–4457. https://doi.org/10.5194/acp-19-4439-2019

Luo, X., Vahmani, P., Hong, T., & Jones, A. (2020). City-Scale Building Anthropogenic Heating during Heat Waves. *Atmosphere*, *11*(11), 1206. https://doi.org/10.3390/atmos11111206

Schlaerth, H. L., Silva, S. J., Li, Y., & Li, D. (2023). Albedo as a Competing Warming Effect of Urban Greening. *Journal of Geophysical Research: Atmospheres*, *128*(24), e2023JD038764. https://doi.org/10.1029/2023JD038764

Stewart, I. D., & Oke, T. R. (2012). Local Climate Zones for Urban Temperature Studies. https://doi.org/10.1175/BAMS-D-11-00019.1

Vahmani, P., & Ban-Weiss, G. (2016a). Climatic consequences of adopting drought-tolerant vegetation over Los Angeles as a response to California drought. *Geophysical Research Letters*, *43*(15), 8240–8249. https://doi.org/10.1002/2016GL069658

Vahmani, P., & Ban-Weiss, G. A. (2016b). Impact of remotely sensed albedo and vegetation fraction on simulation of urban climate in WRF-urban canopy model: A case study of the urban heat island in Los Angeles. *Journal of Geophysical Research: Atmospheres*, *121*(4), 1511–1531. https://doi.org/10.1002/2015JD023718

Vahmani, P., & Hogue, T. S. (2015). Urban irrigation effects on WRF-UCM summertime forecast skill over the Los Angeles metropolitan area. *Journal of Geophysical Research: Atmospheres*, *120*(19), 9869–9881. https://doi.org/10.1002/2015JD023239

Vahmani, P, Sun, F., Hall, A., & Ban-Weiss, G. (2016). Investigating the climate impacts of urbanization and the potential for cool roofs to counter future climate change in Southern California. *Environmental Research Letters*, *11*(12), 124027. https://doi.org/10.1088/1748-9326/11/12/124027

Vahmani, P, Jones, A. D., & Patricola, C. M. (2019). Interacting implications of climate change, population dynamics, and urban heat mitigation for future exposure to heat extremes. *Environmental Research Letters*, *14*(8), 084051. https://doi.org/10.1088/1748-9326/ab28b0

Vahmani, P., Jones, A. D., & Li, D. (2022). Will Anthropogenic Warming Increase Evapotranspiration? Examining Irrigation Water Demand Implications of Climate Change in California. *Earth’s Future*, *10*(1), e2021EF002221. https://doi.org/10.1029/2021EF002221

Vahmani, Pouya, & Jones, A. D. (2017). Water conservation benefits of urban heat mitigation. *Nature Communications*, *8*(1), 1072. https://doi.org/10.1038/s41467-017-01346-1

Vahmani, Pouya, Luo, X., Jones, A., & Hong, T. (2022). Anthropogenic heating of the urban environment: An investigation of feedback dynamics between urban micro-climate and decomposed anthropogenic heating from buildings. *Building and Environment*, *213*, 108841. https://doi.org/10.1016/j.buildenv.2022.108841

Xu, Y., Vahmani, P., Jones, A., & Hong, T. (2024). Anthropogenic heat from buildings in Los Angeles County: A simulation framework and assessment. *Sustainable Cities and Society*, *107*, 105468. https://doi.org/10.1016/j.scs.2024.105468

Zhang, J., Mohegh, A., Li, Y., Levinson, R., & Ban-Weiss, G. (2018). Systematic comparison of the influence of cool wall versus cool roof adoption on urban climate in the Los Angeles basin. *Environmental Science & Technology*, *52*(19), 11188–11197. https://doi.org/10.1021/acs.est.8b00732

Zhang, J., Li, Y., Tao, W., Liu, J., Levinson, R., Mohegh, A., & Ban-Weiss, G. (2019). Investigating the Urban Air Quality Effects of Cool Walls and Cool Roofs in Southern California. *Environmental Science & Technology*, *53*(13), 7532–7542. https://doi.org/10.1021/acs.est.9b00626
